# *DAB2*^+^ macrophages support *FAP*^+^ fibroblasts in shaping tumor barrier and inducing poor clinical outcomes in liver cancer

**DOI:** 10.7150/thno.99046

**Published:** 2024-08-12

**Authors:** Fei Long, Wei Zhong, Faming Zhao, Yaqi Xu, Xin Hu, Gaihua Jia, Lanxiang Huang, Kezhen Yi, Na Wang, Huaqi Si, Jun Wang, Bicheng Wang, Yuan Rong, Yufeng Yuan, Chunhui Yuan, Fubing Wang

**Affiliations:** 1 Department of Laboratory Medicine, Zhongnan Hospital of Wuhan University, Wuhan, China.; 2 Center for Single-Cell Omics and Tumor Liquid Biopsy, Zhongnan Hospital of Wuhan University, Wuhan, China.; 3 Department of Laboratory Medicine, Wuhan Children's Hospital (Wuhan Maternal and Child Healthcare Hospital), Tongji Medical College, Huazhong University of Science and Technology, Wuhan, China.; 4 Department of Pathology, Zhongnan Hospital of Wuhan University, Wuhan, China; 5 Department of Hepatobiliary and Pancreatic Surgery, Zhongnan Hospital of Wuhan University, Wuhan, China; 6 Wuhan Research Center for Infectious Diseases and Cancer, Chinese Academy of Medical Sciences, Wuhan, China.

**Keywords:** *FAP*^+^ fibroblast, *DAB2*^+^ macrophage, immune exclusion, single-cell RNA sequencing, prognosis

## Abstract

**Background:** Cancer-associated fibroblasts (CAFs) are the key components of the immune barrier in liver cancer. Therefore, gaining a deeper understanding of the heterogeneity and intercellular communication of CAFs holds utmost importance in boosting immunotherapy effectiveness and improving clinical outcomes.

**Methods:** A comprehensive analysis by combing single-cell, bulk, and spatial transcriptome profiling with multiplexed immunofluorescence was conducted to unravel the complexities of CAFs in liver cancer.

**Results:** Through an integrated approach involving 235 liver cancer scRNA-seq samples encompassing over 1.2 million cells, we found that CAFs were particularly increased in hepatocellular carcinoma (HCC) and intrahepatic cholangiocarcinoma (ICC). *FAP*^+^ fibroblasts were identified as the dominant subtype of CAFs, and which were mainly involved in extracellular matrix organization and angiogenesis. These CAFs were enriched in the tumor boundary of HCC, but diffusely scattered within ICC. The *DAB2*^+^ and *SPP1*^+^ tumor-associated macrophages (TAMs) reinforce the function of *FAP*^+^ CAFs through signals such as TGF-β, PDGF, and ADM. Notably, the interaction between *DAB2*^+^ TAMs and *FAP*^+^ CAFs promoted the formation of immune barrier and correlated with poorer patient survival, non-response to immunotherapy in HCC. High FAP and DAB2 immunohistochemical scores predicted shorter survival and higher serum AFP concentration in a local clinical cohort of 90 HCC patients. Furthermore, this communication pattern might be applicable to other solid malignancies as well.

**Conclusions:** The interaction between *DAB2*^+^ TAMs and *FAP*^+^ CAFs appears crucial in shaping the immune barrier. Strategies aimed at disrupting this communication or inhibiting the functions of *FAP*^+^ CAFs could potentially enhance immunotherapy effectiveness and improve clinical outcomes.

## Introduction

Liver cancer ranks as the third leading cause of cancer-related deaths and the sixth most prevalent tumor type globally 1. Hepatocellular carcinoma (HCC) is the most common liver cancer, followed by intrahepatic cholangiocarcinoma (ICC), which together account for more than 95% of the cases 2. In 2020, the U.S. Food and Drug Administration (FDA) approved the combination of atezolizumab (anti-PD-L1) and bevacizumab (anti-VEGF) as a first-line treatment for advanced HCC, which demonstrated superior efficacy to sorafenib in the phase III IMBRAVE-150 study (NCTO3434379) 3. However, despite these remarkable breakthroughs, immunotherapy proves effective only for 20-30% of patients 4. The path to improved immunotherapy response is largely limited by the heterogeneity of the liver cancer microenvironment.

In recent years, there has been a surge of interest in stromal cells, largely driven by advancements in single-cell RNA sequencing (scRNA-seq) and spatial transcriptome (ST) technology. Fibroblasts, the most prevalent stromal cell type present in the tumor microenvironment (TME), play a crucial role in regulating the infiltration and function of anti-tumor immune cells via emerging as key players in TME remodeling 5. Furthermore, they promote tumor cell growth, metastasis, and drug resistance through various pathways. Tumor-promoting fibroblasts, known as cancer-associated fibroblasts (CAFs), provide a fertile environment for cancer cells, akin to "soil" for cancer "seeds". Tumors are often likened to "unhealable wounds", and CAFs respond to this perceived tissue damage by attempting to repair it, leading to their significant accumulation 6.

Multiple markers for CAFs have been identified, such as αSMA, FAP, periostin (POSTN), PDGFRα/β, and FSP-1 7. Interestingly, cell clusters identified by these markers exhibit different expression profiles, indicating a high degree of heterogeneity among CAFs within the TME. ScRNA-seq and multiplex imaging techniques have been employed to delineate various CAF subtypes across a range of cancers. Chhabra* et al.* have categorized CAFs into four main groups: inflammatory CAF (iCAF), myofibroblastic CAF (myCAF), antigen-presenting CAF (apCAF), and vascular CAF (vCAF) 8. Conversely, Zhu* et al.* identified five major CAFs based on gene expression and function from scRNA-seq in HCC 9, while Zhang* et al.* recognized four major CAFs in ICC 10. The heterogeneity of CAFs persists across different tumor contexts, necessitating a high-resolution, comprehensive assessment of CAF subtypes in larger cohorts, particularly focusing on their function and intercellular interactions.

An Achilles' heel of current chemotherapy and immunotherapy approaches is that most current therapies target the rapidly proliferating tumor "seeds," yet largely ignore the contribution of fibroblasts or the fertilizing tumor "soil". The extracellular matrix (ECM) generated by CAFs undergoes active remodeling and degradation, promotes tumor progression, which is largely mediated by tumor associated macrophages (TAMs) and matrix metalloproteinases 11. The balance between ECM production and degradation not only affects cell migration and immune cell infiltration but also regulates cell signaling processes. ECM receptors (e.g., CD44, integrins, and discoidin domain receptors) and ECM ligands (e.g., cleaved forms of collagen) play a crucial role in this remodeling during tumorigenesis 12, 13. The secretion of increased and reorganized ECM proteins by CAFs leads to enhanced fibrillar collagen deposition, which results in ECM stiffening, further promoting tumor cell proliferation and invasion 14. CAFs and TAMs have been shown to have extensive cellular interactions in various tumors, including liver cancer. They are closely associated with ECM remodeling, promote the formation of pro-tumor connective tissue, inhibit lymphocyte infiltration, and correlate with patients' non-response to immunotherapy 15-17. However, the heterogeneous subtypes of CAFs and TAMs in liver cancer remain poorly characterized, and further research is needed to determine the communication and functional impact between these tumor-infiltrating cells.

Here, we unveil the heterogeneity and interactions of CAFs and TAMs across different liver cancer subtypes, highlighting the intricacy of studying cellular interactions and distribution patterns within distinct spatial regions to comprehend the TME. This understanding is crucial for elucidating the reasons behind immunotherapy resistance and enhancing the clinical outcomes for patients.

## Methods

### Data retrieval and preprocessing

A single-cell RNA sequencing (scRNA-seq) discovery cohort comprising 156 samples and corresponding clinical data from 120 liver cancer patients was provided by Xue *et al.* 18 (**Table S1**). This included 14 adjacent liver (AL), 82 hepatocellular carcinoma (HCC), 31 intrahepatic cholangiocarcinoma (ICC), 9 combined hepatocellular and cholangiocarcinoma (CHC), 10 secondary liver cancer (SLC), and 10 peripheral blood (PB) samples. The unprocessed FASTQ data for these samples (BioProject ID: PRJCA007744) is stored at the China National Center for Bioinformation (CNCB) (https://www.cncb.ac.cn/). Furthermore, we obtained scRNA-seq data of 79 liver cancer samples as a validation cohort from Gene Expression Omnibus (GEO) (GSE151530, GSE125449) and CNCB (PRJCA002164) 19-21. The datasets included 7 AL samples, 48 HCC samples and 24 ICC samples. The single-cell FASTQ data were filtered and aligned using CellRanger (v.3.0.1, 10 × Genomics) with the human reference genome GRCh38. Adjacent-tumor, tumor, and tumor leading edge spatial transcriptome (ST) sequencing data of 12 cases of HCC and one case of ICC were obtained from the study of Wu *et al.* 22. The ST data of the three HCC cases that contained information on the response to immunotherapy were obtained from the study of Liu *et al.* 15. The authors obtained bulk RNA-seq or microarray data for 1518 cases of HCC and ICC from seven independent cohorts of cancerous and paracancerous samples. These included the TCGA-LIHC/TCGA-CHOL (n = 469), GSE14520 (n = 488), GSE40873 (n = 49), GSE76427 (n = 167), GSE116174 (n = 64), GSE54236 (n = 161), and GSE89749 (n = 120). Furthermore, RNA-seq data from 298 tumor samples from the IMvigor210 project that received atezolizumab immunotherapy were incorporated into this study 23. The normalized pan-cancer bulk RNA-seq data and patient clinical data were obtained from UCSC-Xena (https://xenabrowser.net/datapages/) (PANCAN cohort) and included 39 cancer types and 11,060 samples. Pan-cancer scRNA-seq data were mainly derived from the study of Luo *et al.* (GSE210347) 24, which included 10 solid tumor types and 226 samples. Due to the lack of HCC samples, the 79 samples from the validation cohort were integrated for analysis, and corrections for different batches of cohorts were performed using the R package harmony. Pan-cancer ST sequencing data were obtained from GEO, European Genome-phenome Archive (EGA), Genome Sequence Archive (GSA), Mendeley Data, and 10x, including skin cutaneous melanoma (SKCM, GSM5420750), breast cancer(BRCA, Human Breast Cancer: Visium Fresh Frozen, Whole Transcriptome from 10x), lung adenocarcinoma (LUAD, GSM5420751), renal cell carcinoma (RCC, GSM5924036), medulloblastoma (MB, EGAS00001006124), pancreatic ductal adenocarcinoma (PDAC, GSM6505134), squamous cell carcinoma (SCC, V10F24_015_A1, doi.org/10.17632/2bh5fchcv6.1), ependymoma (EPN, GSM5844724), colorectal cancer (CRC, P6, HRA000979), head and neck squamous cell carcinoma (HNSC, GSM5494476), ovarian cancer (OV, Human Ovarian Cancer, 11 mm Capture Area from 10x), prostate adenocarcinoma (PRAD, EGAS00001006124), gastrointestinal stromal tumor (GIST, GSM6177607). Finally, we integrated renal cell carcinoma (RCC) (SCP1288, Single Cell Portal, R = 2, NR = 3, UT = 3, NE = 1) 25, non-small cell lung cancer (NSCLC) (GSE207422, R = 9, NR = 6) 26, basal cell carcinoma (BCC) (GSE123813, R = 6, NR = 4) 27 of the scRNA-seq immunotherapy cohort, corrected for different batches of cohorts using R package harmony.

### Single-cell data analysis

ScRNA-seq data were initially filtered by the R package Seurat to retain cells that met the following criteria: (1) the number of detected genes was above 500 and below 6000; (2) the number of UMI count per cell lower than 30,000; and (3) the percentage of mitochondrial genes lower than 50%. Subsequently, the data underwent normalization and scaling, with the top 2000 highly variable genes (HVGs) calculated by the FindVariableFeatures function. HVGs were employed for principal component analysis (PCA), with 50 principal components selected for subsequent cell clustering. Furthermore, the R package harmony was utilized to remove batch effects between cohorts in the validation set. In order to reflect the true level of cellular heterogeneity, a batch correction was not applied to the discovery set, which originated from the same cohort. Cell subclusters were acquired using the FindNeighbors and FindClusters functions, with resolution set to 1 for all main celltypes and 0.3 for fibroblasts and macrophages. Finally, cell visualization was performed using uniform manifold approximation and projection (UMAP). The cell annotations were primarily based on previous studies 18, 24.

### Cell preference analysis

To ascertain the infiltration intensity of different cell types in disparate samples, we delineated cellular preferences through the application of the odds ratios (OR) algorithm, as detailed by Zheng *et al.* 28. In this context, an OR value exceeding 1.5 signified a cell's significant enrichment in the designated class of samples, whereas an OR value below 0.5 indicated a cell's significant depletion (BH-corrected *P* < 1e-10).

### Functional analysis of cell subclusters

In order to gain insight into the functional characterization of different cell subclusters, the highly expressed genes of cell subclusters (adj. *P* < 0.05, log2FC > 0.5) were initially obtained by the FindAllMarkers function in Seurat. This was followed by a functional enrichment analysis based on the GO database, which was conducted using the R package clusterProfiler 29. The entries with *FDR* < 0.05 were considered to be significantly enriched. Furthermore, the UCell scores of 50 key cancer hallmark gene sets from MSigDB (https://www.gsea-msigdb.org/gsea/msigdb/) and specific cell-related pathways in different cell subclusters or sample types were evaluated using the R package irGSEA 30. To further explore the association of cell subclusters with metabolic pathways, the relative enrichment of different metabolic pathways in different cell subclusters was assessed using the R package scMetabolism 31.

### Survival analysis

To assess the association between fibroblast subcluster and patient survival, we employed the relative proportions of fibroblast subclusters as a variable in conjunction with survival data from the single-cell discovery cohort **(Table S2)**. A total of 120 patients with liver cancer were included, of which 2 patients were excluded because they did not have corresponding overall survival information, and 118 patients were finally included, including 7 patients with CHC, 79 patients with HCC, 25 patients with ICC, and 7 patients with SLC. When performing the calculation of cell proportions for individual patients, since multiple tissue samples may be taken from a single patient, we only included the corresponding cancer tissues, and if duplicate samples existed, the average of the cell proportions was taken as the proportion of a particular type of cell for that patient. We then performed univariate Cox risk analyses using the R package survival. Kaplan-Meier (KM) curves were also utilized for survival analysis of selected cell subclusters, with a Log-rank and Cox p-value of less than 0.05 indicating a statistically significant association between the cell subclusters and patient survival. Concurrently, we endeavored to identify cells associated with survival by integrating TCGA bulk RNA-seq and corresponding survival data, linking gene expression and cell expression in bulk via the R package Scissor 32, integrating phenotypes into a network regularized sparse regression model, and identifying Scissor^+^ cells that were hypothesized to be associated with poorer survival of patients. The identified Scissor^+^ cells were considered to be associated with worse patient survival. To assess the association of cells with patient survival in other bulk cohorts, cell type-specific genes (mu = 1) were initially screened using the R package COSG 33. The top 30 ranked genes were then utilized for ssGSEA cell scoring of the bulk samples. This was followed by the calculation of optimal grouping cutoff values and survival analysis for each cohort, conducted using the R package survminer.

### Inference of cell differentiation trajectories

In order to gain insight into the potential cellular differentiation pathways, this study employed the R packages Monocle2 and velocyto.R to perform the corresponding cell trajectory analysis. In particular, RNA velocity analysis was conducted based on loom files generated by transforming bam data from the single-cell validation cohort PRJCA002164. This analysis distinguished between spliced and unspliced transcripts in order to assess changes in expression dynamics. RNA velocity values for genes in each cell were calculated using the R package velocyto.R. RNA velocity vectors were embedded in the UMAP low-dimensional space.

### Spatial transcriptome (ST) analysis

The R package stCancer was employed to perform pre-quality control (QC), clustering, and gene expression analysis on the downloaded ST data. To assess the spatial distribution of the cell subclusters identified from the single-cell cohort, the ST and scRNA-seq expression matrices were first merged and co-dimensioned using the R package CellTrek 34. This was followed by the generation of a sparse graph using a random forest model, and then constructed a spot-cell similarity matrix for the single cells, added spatial coordinate information. The co-localization patterns among different cell subpopulations were summarized using the scoloc function, and the strength of cell co-expression was assessed by Kullback-Leibler divergence (KLD). A higher KLD value indicates a stronger co-localization of cell subclusters. It is important to note that the mapping of single cells to space in HCC and ICC was performed separately, as their tumor environments are distinct. Additionally, we conducted deconvolution analysis using the R package CARD 35 to identify large subclusters of cells that co-localize spatially and perform distributional correlation analysis. In order to distinguish the malignant region (Mal), boundary (Bdy), and non-malignant region (nMal), we employed the R package Cottrazm 36 for region identification and evaluated the cell enrichment scores of different regions based on the previously reported deconvolution approach 37. Furthermore, we conducted a Wilcoxon test to compare the cell enrichment of different spatial regions.

### Immunofluorescence (IF) staining

Seven formalin-fixed paraffin-embedded (FFPE) tissue samples from two HCC and one ICC patients were collected from Zhongnan Hospital of Wuhan University, including two tumor tissues, tow paracancer tissues and three tumor boundary tissues. The study protocol was approved by the Research Ethics Committees of Zhongnan Hospital of Wuhan University. For multiplex IF staining, FFPE tissue sections were cut at a thickness of 5 mm, followed by dewaxing in three changes of xylene for 15 min and 100% ethanol for 5 min, 85% alcohol for 5 min, 75% alcohol for 5 min, and washed by distilled water. Antigen was retrieved at 98 °C for 10 min in 10 mM citrate buffer pH 6. The sections were placed in a 3% hydrogen peroxide solution and incubated for 25 minutes at room temperature in the dark to allow endogenous peroxidase to be sealed. Subsequently, the slides were placed in phosphate-buffered saline (PBS, pH 7.4) on a decolorizing shaker and washed by shaking three times for five minutes each time. Subsequently, 10% goat serum was added and incubated for 30 minutes at room temperature. Slides were next stained with DAB2 (1:200; Proteintech Cat# 10109-2-AP, RRID:AB_2089700) , CD68 (1:1000, Abcam Cat# ab955, RRID:AB_307338), SPP1 (1:200; PB0589, BOSTER) or FAP (1:200, Bioss Cat# bs-5758R, RRID:AB_11072621) antibodies and incubated flat in a wet box at 4°C overnight. The slides were then incubated with secondary antibodies (1:400 for each; HRP-anti-rabbit IgG, SeraCare KPL Cat# 5220-0336, RRID:AB_2857917; or HRP-anti-mouse IgG, SeraCare KPL Cat# 5220-0341 (also 074-1806), RRID:AB_2891080) for 50 min at room temperature. Following each staining cycle, the TSA reagent (Baiqiandu; Tyramine-CY3, 1:500, B30001; Tyramine-488, 1:200, B30002; Tyramine-CY5, 1:2000, B30003; Tyramine-594, 1:500, B30004) was added, incubated at room temperature for 20 minutes, and the slides were subjected to 1× citric acid repair solution. This was then repaired in a microwave oven on high for 6 minutes, cooled down to room temperature, and incubated dropwise with 10% goat serum for 10 minutes at room temperature. The samples were counterstained for nuclei with DAPI for 10 min and mounted in mounting medium. Multispectral images were scanned with SQS40R.

### Bulk deconvolution analysis

To assess the infiltration correlation of different cell subclusters in bulk RNA-seq and microarray data, the cell abundance in each sample was first estimated using CIBERSORTx 38. This was done in two stages. Firstly, since the amount of single-cell data was more than 1 million, we randomly sampled each cell type, retained 1,000 cells for each cell type, and derived a single-cell count expression matrix to create the signature matrix. Secondly, the constructed signature matrix and the bulk-based mixture file were utilized for CIBERSORTx analysis, with quantile normalization disabled during the processing of RNA-seq data. The permutations for significance analysis were set to 100. Following the calculation of the cell infiltration abundance for each sample, the strength of the association between different cells was evaluated using Spearman correlation analysis. A correlation was considered significant if the p-value was less than 0.05 and the absolute value of the correlation coefficient was greater than 0.2.

### Cell communication analysis

The R package nichenetr 39 was employed to infer potential communication between macrophages and fibroblasts. To gain further insight into the functional impact of macrophages on *FAP* ^+^ fibroblasts in different tumor types (HCC and ICC) and to identify ligands and receptors for communication, we defined *FAP* ^+^ CAF as the receiver, other subtypes of macrophages as the sender, the reference condition set as ICC, and the condition set as HCC. Subsequently, the top 30 ligands, receptors and their target genes, ranked according to aupr_corrected, were used for heat map visualization. Next, the association of each ligand or receptor genes with the risk of patients were evaluated using univariate Cox analysis based on the TCGA-LIHC cohort. Functional enrichment analysis was conducted on all target genes or selected genes via the g:Profiler tool 40 with background databases comprising GO, KEGG, Reactome, and WikiPathways. The method g:SCS was selected for p-value correction, and pathways with a corrected p-value of less than 0.05 were considered significantly enriched. To assess the overall impact of *FAP* ^+^ fibroblasts on downstream cells, the R package CellChat 41 was utilized to analyze the overall outgoing signals and regulatory pathways of *FAP* ^+^ fibroblasts. Furthermore, we identified ligand-receptor communication between *FAP* ^+^ fibroblasts and endothelial and tumor cells by CellphoneDB 42 in ICC, which suggests their direct communicative role in the ICC environment.

### Quantifying communication between TAM and *FAP* ^+^ CAF

The top 30 ligand-receptor pairs obtained through NicheNet analysis, sorted by aupr_corrected, were used for cellular communication quantification. Since a ligand may correspond to multiple receptors, a total of 90 ligand and receptor genes were obtained. The ssGSEA score, designated LRscore, was calculated by first performing ssGSEA based on 90 genes in distinct bulk cohorts, followed by the determination of optimal group cutoff values and survival analysis for each cohort via the R packages survminer and survival.

### Potential drug prediction

The 50% inhibitory concentration (IC50) of 198 targeted drugs in TCGA-LIHC patients was initially estimated using the R package oncoPredict 43 based on GDSC. Drugs associated with LRscore were identified by Spearman's correlation analysis, and those with *P* > 0.05 were filtered out. Furthermore, we predicted drugs that block the communication between TAM and *FAP* ^+^CAF using the R package sc2MeNetDrug 44. After obtaining the up-regulated genes for each cell cluster in the cell-cell communication section, we applied GSEA and drug hierarchy matrices to identify potential drugs. Drug clustering was performed based on the SMILES information of the drug chemical structure, with drugs with high similarity clustered together and potentially playing similar roles.

### Immunohistochemistry staining

Tissue microarrays (Shanghai Outdo Biotech, Shanghai, China) consisting of 90 HCC boundary, 10 ICC tumors, 8 HCC tumors, and 8 matched adjacent normal tissues. Firstly, an endogenous peroxidase blocker was added dropwise to the tissue microarrays, incubated at room temperature for 10 minutes, and washed with double-distilled water to inactivate endogenous peroxidase. Subsequently, antigen repair was performed by placing the microarrays into EDTA antigen repair solution, heating on high for 8 minutes in a microwave oven, cooling naturally for 8 minutes, then heating on high for 8 minutes, and cooling to room temperature. The 5% BSA blocking solution was added, incubated at 37°C for 30 minutes, and the excess liquid was shaken off. The diluted primary antibody, either FAP (1:50, BM5121, Boster) or DAB2 (1:50, Proteintech Cat# 10109-2-AP, RRID:AB_2089700), was added and incubated at 4°C overnight. Following removal, the cells were rewarmed at 37°C for 30 minutes and washed with PBS (pH 7.2-7.6) for 5 minutes three times. The HRP-anti-rabbit IgG (SV002, Boster) was added, incubated at 37°C for 30 minutes, and washed with PBS (pH 7.2-7.6) for 5 minutes three times. The reaction time was controlled by adding drops of DAB working solution and observing under the microscope. Major hematoxylin was added and incubated at room temperature for one minute, after which the slices were washed with PBS (pH 7.2-7.6). Finally, the slices were sealed with neutral gum and scanned with SQS40R to obtain images.

### Immunotherapy correlation analysis

In order to evaluate the effect of *FAP* ^+^ CAF and *DAB2* ^+^ TAM on the immunotherapy response of tumor patients, we first predicted the immune response grouping of TCGA-LIHC samples by the TIDE algorithm. We then grouped the samples based on the CIBERSORTx-imputed proportions of *FAP* ^+^ CAF and *DAB2* ^+^ TAM cells, and observed the proportions of grouped samples in the immune therapy responsive and non-responsive groups. Finally, inter-group clustering consistency was calculated using the SubMap 45 algorithm. Furthermore, we conducted ssGSEA on samples for *FAP* ^+^ CAF and *DAB2* ^+^ TAM based on the Imvigor210 immunotherapy cohort, and employed the Wilcoxon test to assess the statistical significance between responding and non-responding groups. The *FAP* ^+^CAF-related genes included *VCAN*, *FAP*, *COL8A1*, *THBS2*, *NTM*, *POSTN*, *INHBA*, *ISLR*, *COL5A1*, *COL6A3*, *MMP2*, *LTBP2*, and *LOXL1*. *DAB2* ^+^TAM-related genes included *MAF*, *A2M*, *DAB2*, *GPR34*, *CD209*, *GYPC*, *FOLR2*, *SLC40A1*, and *IGF1*. Finally, scRNA-seq data derived from three independent cohorts containing information on response to immunotherapy were integrated to analyze the relative proportions of *FAP* ^+^ CAF and *DAB2* ^+^ TAM in different response groups.

### Statistical analysis

Statistical analyses and visualization were conducted using R (v. 4.3.0) and Sangerbox (http://sangerbox.com/login.html). The differences between the two groups were compared using the Wilcoxon test, and the paired samples were assessed for significance using the paired Student's t-test, unless otherwise specified. Survival analysis was conducted using the Kaplan-Meier method, and statistical significance was assessed using the Log-rank test. The median value or the best cut-off value was used as the cut-off point. The *P* < 0.05 was considered to be statistically significant. Correlation analyses were conducted using the Spearman method, given that the data were non-normally distributed. Further statistical details can be found in the figure legends.

## Results

### Increased fibroblasts and macrophages in HCC and ICC

**Figure 1:**
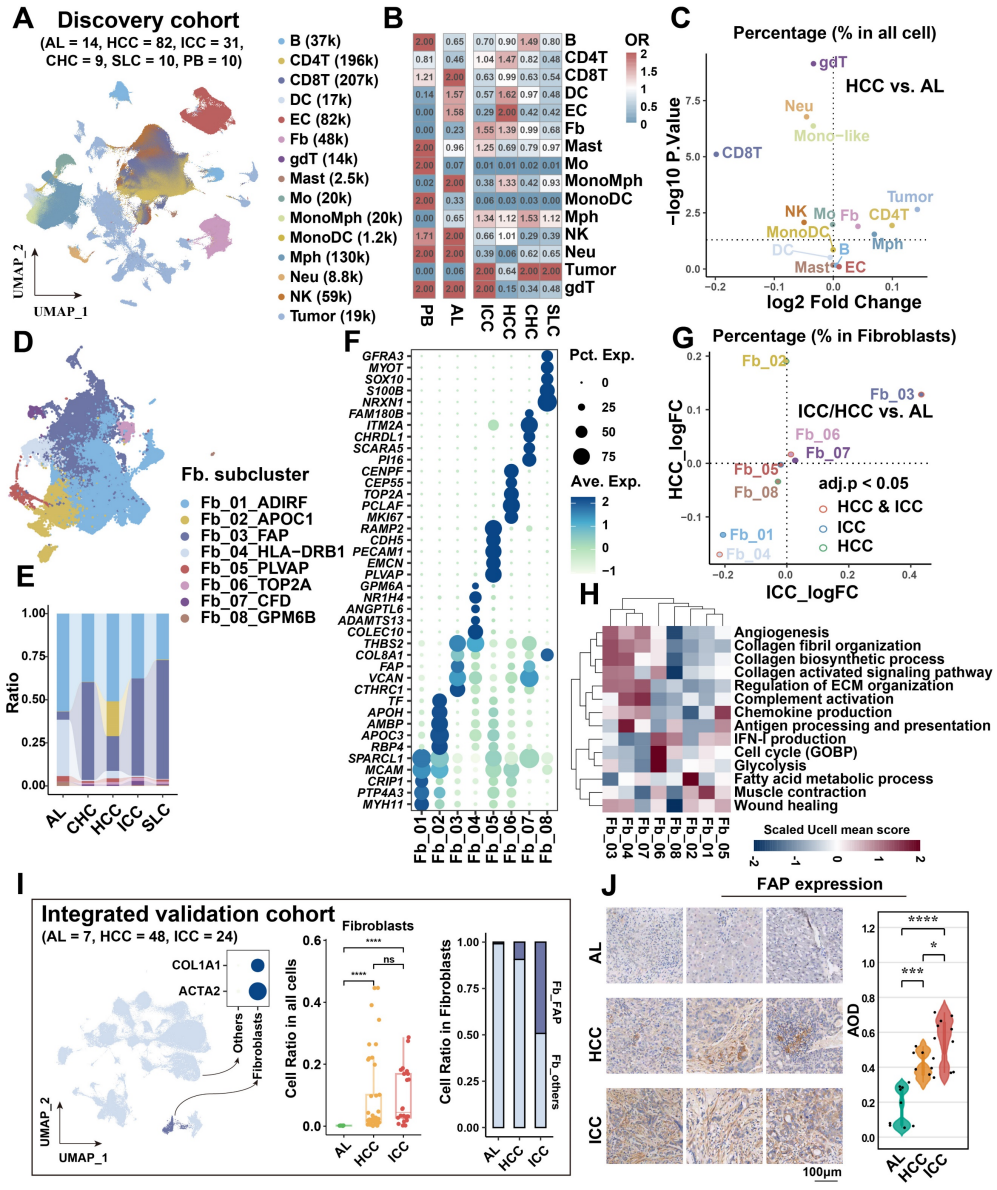
**Identification of key cancer-associated fibroblasts.** (A) UMAP shows the cellular clustering of major cell types in the discovery cohort and the corresponding cell numbers. (B) Heatmap showing the cell preference of different major cell types in peripheral blood (PB), adjacent liver (AL), intrahepatic cholangiocarcinoma (ICC), hepatocellular carcinoma (HCC), combined hepatocellular and cholangiocarcinoma (CHC), and secondary liver cancer (SLC). (C) Volcano plot showing the difference in the proportion of major cell types in HCC (n = 82) versus AL (n = 14). (D) UMAP shows the distribution of fibroblast subtypes in the discovery cohort. (E) Stacking plot shows the percentage of fibroblast subtypes in the five tissue types. (F) Dot plot shows top5 highly expressed genes for each fibroblast subtypes. (G) Volcano plot comparing the relative abundance of HCC/ICC versus AL fibroblast subtypes. (H) Heatmap showing the Ucell enrichment scores of key biological entries of fibroblasts in different subtypes. (I) UMAP shows the identification of fibroblasts from an integrated validated single-cell cohort (left); box plot shows the significantly higher relative abundance of fibroblast in AL, HCC and ICC (median); stacking plot shows the progressively higher proportion of *FAP* ^+^ CAF in HCC and ICC compared to AL (right). (J) Immunohistochemically stained pathology sections and box plot showed progressively higher FAP expression and average optical density (AOD) in AL (n = 8), HCC (n = 8) and ICC (n = 10) samples.

To elucidate the cellular composition across various liver cancer types and sample sources, we included 156 scRNA-seq samples as a discovery cohort from the study of Xue *et al* 18*.* The discovery cohort encompassed 14 adjacent liver (AL), 82 HCC, 31 ICC, 9 combined hepatocellular-cholangiocarcinoma (CHC), 10 secondary liver cancer (SLC), and 10 peripheral blood (PB) samples. After standard cell filtering and processing, a total of 1,034,073 cells were obtained, of which 83,154 were from AL, 606,191 from HCC, 174,853 from ICC, 46,210 from CHC, 59,077 from SLC, and 64,588 from PB. After further clustering and visualization, we obtained a total of 15 major cell types** (Figure 1A, Figure S1A & S1B)**: B cell (n = 36,685) marked by *CD79A*, *MS4A1*, and *IGHM*; CD4^+^ T (n = 196,129) marked by *CD3D* and *CD4*; CD8^+^ T (n = 206,952) marked by *CD3D* and *CD8A*; dendritic cell (DC, n = 17,369) marked by *CD1C*, *CLEC9A* and *LAMP3*; endothelial cell (EC, n = 82,342) marked by *CDH5* and *VWF*; fibroblast (Fb, n = 48,286) marked by *ACTA2* and *COL1A2*; γδT (gdT, n = 13,760) marked by *CD3D*, *TRGC2* and *TRDC*; mast cell (n = 2,459) marked by *TPSAB1* and *MS4A2*; monocyte (Mo, n = 19,755) marked by *FCN1*, *CD14*, and *FCGR3A*, derived from PB; mono-like macrophage (MonoMph, n = 20,191) marked by *FCN1* and *C1QB*, derived from tissue; mono-like DC (MonoDC, n = 1223) marked by *FCN1* and *CD1C*; macrophage (Mph, n = 129,766) marked by *C1QC*, *C1QB*, and *CD68*; neutrophil (Neu, n = 59,099) marked by *FCGR3B* and *CSF3R*; natural killer cell (NK, n = 59,099) marked by *KLRF1* and *NKG7*; tumor cell (n = 191,282) marked by *EPCAM*, *ALB*, and *APOA2*. The sample distribution revealed that tumor cells exhibited the highest heterogeneity, aligning with previous study 46 **(Figure S1B)**. Subsequently, we conducted a comparative analysis of the infiltration of major cell types across different sample types. Our findings revealed that the cell preference and proportion of fibroblasts and macrophages were generally elevated in all liver cancer types compared with AL **(Figure 1B, Figure S1C)**. Specifically, fibroblasts were most highly enriched in the two main liver cancer types, HCC and ICC** (Figure 1B)**. Significant increases in fibroblasts and macrophages were observed in both HCC and ICC compared to AL (BH-corrected *P* < 0.05) **(Figure 1C, Figure S1G)**. These findings indicate that fibroblasts and macrophages may play a pivotal role in shaping the pro-tumor microenvironment in liver cancer, particularly in HCC and ICC.

### *FAP* ^+^ fibroblasts are the dominant tumor-associated fibroblasts (CAFs)

To gain further insight into the pivotal role of fibroblasts in shaping the microenvironment of liver cancer, we referenced previously reported fibroblast subtype-specific markers and categorized them into eight clusters (Fb_01_ADIRF, Fb_02_APOC1, Fb_03_FAP, Fb_04_HLA-DRB1, Fb_05_PLVAP, Fb_06_TOP2A, Fb_07_CFD, Fb_08_GPM6B) 24** (Figure 1D, Figure S1D & S1E, Table S3).** In comparison to AL, Fb_03_FAP was observed to be elevated in all liver cancer types, representing about 50% of all fibroblasts **(Figure 1E, Figure S1F)**. Interestingly, Fb_02_APOC1 was predominantly enriched in HCC, possibly due to the unique metabolic milieu in HCC. In contrast, Fb_04_HLA-DRB1 was highest in the AL, expressing typical hepatic stellate cell markers (*LRAT*, *HGF*, and *RELN*) 47, 48 **(Figure 1E, Figure S1E)**.

To elucidate the functions of these distinct subtypes of fibroblasts, we conducted a functional enrichment analysis based on the highly expressed genes of each cell subcluster **(Figure 1F, Figure S2A)**. Fb_01 was predominantly enriched in pathways related to the muscle system and muscle contraction** (Figure 1F)**, expressed high levels of *ACTA2*, *ADIRF*, and *MYH11*, similar to smooth muscle cells (SMCs) or myofibroblasts 24, 49. Fb_02 was found to be enriched in lipid-related pathways, with high expression of apolipoprotein genes *APOC1* and *APOC3*. This is similar to the lipid processing CAFs (lpCAFs) 9, with the highest overall metabolic scores and demonstrated higher fatty acid degradation, beta-alanine metabolism, valine, and other fatty acid or amino acid metabolism-related entry scores **(Figure S2B).** Fb_03 belongs to matrix-associated fibroblasts 49, which was found to be significantly enriched in extracellular matrix organization and collagen fibril organization, with high expression of fibroblast activation protein-related gene *FAP*, extracellular matrix-related marker *VCAN*, and *COL1A1*. These cells are significantly characterized by hypoxia pathway activation and D-glutamine and D-glutamate metabolism activation **(Figure S2B & S2C)**. Additionally, Fb_07 exhibits a comparable expression profile to Fb_03, yet is enriched in complement-related pathways and exhibits high expression of the complement-related gene *CFD*, which may belong to a subclass of *FAP* ^+^ CAF. Fb_04 is involved in antigen processing and presentation, with high expression of the MHC class II molecule *HLA-DRB1* and the chemokine *CXCL12*. It is classified as a classical apCAF 9. *PLVAP*, a classical endothelial cell-associated marker, is highly expressed in Fb_05, enriched in the endothelium development, and endothelial cell differentiation pathways, suggesting its potential endothelial cell origin through endothelial-mesenchymal transition, categorized as CAF_EndMT_ in the study of Luo *et al.* 24. Fb_06 was characterized by high proliferation and belonged to cycling CAF. Additionally, Fb_08 was enriched to nerve-related pathway with high expression of *S100B* and *GPM6B*, which belonged to fibroblast-like peripheral nerve cells, which was also found in Luo *et al.*'s study, but this type of cells was mainly enriched in colorectal cancer and suggests the impact of perineural invasion on patient risk 24.

We further evaluated the infiltration differences of fibroblast subtypes in HCC and ICC. Our findings revealed that *FAP* ^+^ CAFs were significantly increased in both HCC and ICC compared to AL (BH-adjusted *P* < 0.05) **(Figure 1G)**. Notably, the infiltration of *FAP*^+^ CAFs was higher in ICC compared to HCC (BH-adjusted *P* < 0.05) **(Figure S1H)**.

Key characteristics of *FAP*^+^ CAFs included highly activated angiogenesis, collagen fibril organization, and collagen biosynthetic processes **(Figure 1H)**. A comparison of *FAP*^+^ CAFs across sample types showed that glycolysis and angiogenesis were most enriched in ICC, while the collagen-activated signaling pathway was activated in both HCC and ICC **(Figure S1I)**. These results indicate that *FAP* ^+^ CAFs may have partial functional preferences in different types of liver cancer.

To further validate the stable increase of *FAP*^+^ CAFs in the tumor microenvironment, we integrated a single-cell validation cohort comprising 79 samples 19, 50, and which demonstrated a gradual increase in the proportion of *FAP* ^+^ CAFs from AL, HCC, to ICC **(Figure 1I)**. After deconvolution single cells into the bulk cohort (TCGA-LIHC/CHOL) as well as in tumor-AL paired scRNA-seq samples, a significant increase of *FAP* ^+^ CAFs ratio in liver cancer samples was observed (Wilcoxon test, *P* < 0.01; paired t-test, *P* < 0.05)** (Figure S1J)**. Immunohistochemical images also confirmed elevated FAP expression in HCC and ICC samples **(Figure 1J)**. Collectively, the augmented abundance of *FAP* ^+^ CAFs emerges as a widespread phenomenon in tumors, despite potential functional diversity across various liver cancer subtypes.

### Increased *FAP* ^+^ CAFs suggests tumor progression

**Figure 2:**
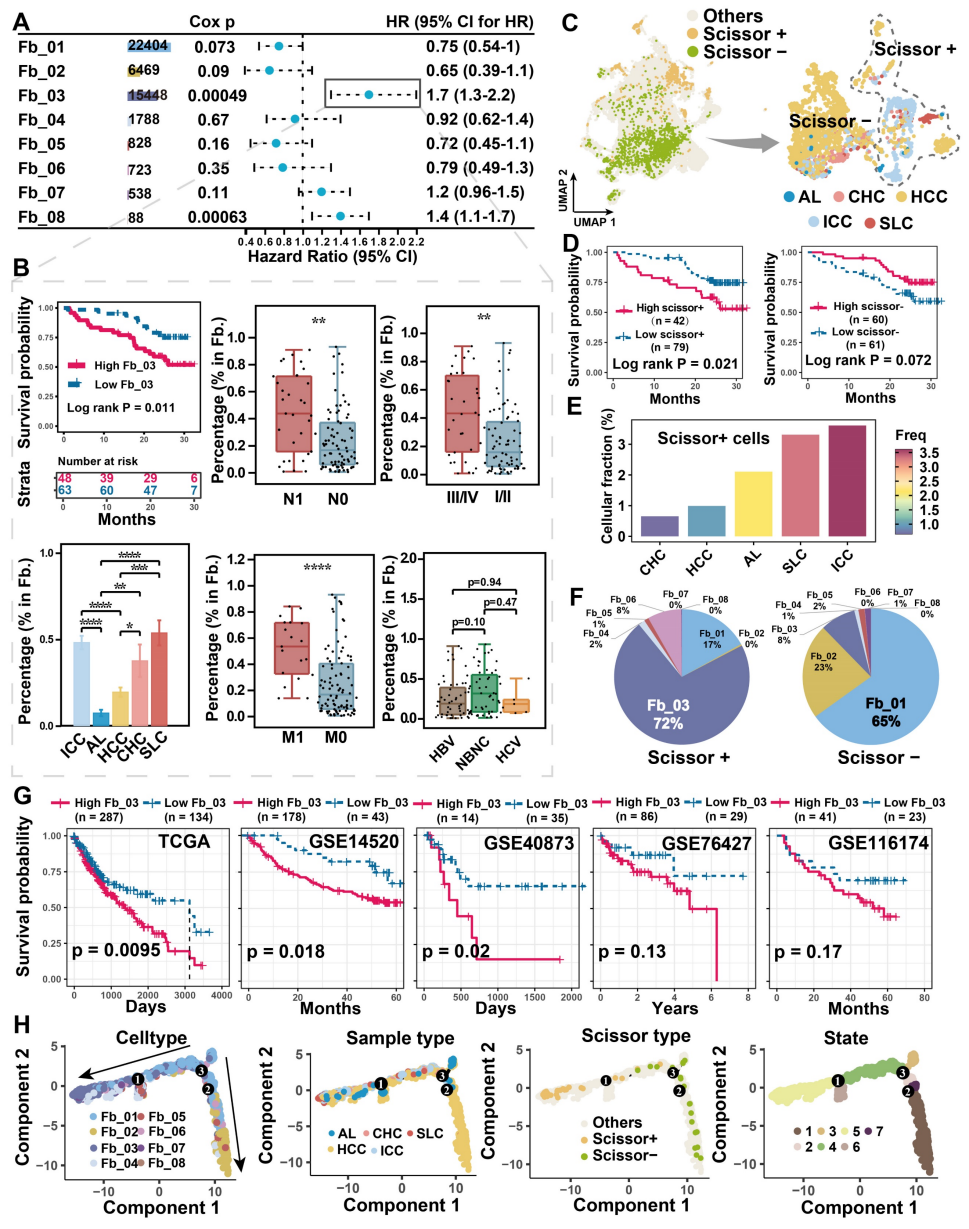
** Association of *FAP* ^+^ CAF with clinical features and differentiation origins.** (A) Forest plot showing the proportion of *FAP* ^+^ CAF in the single-cell discovery cohort was significantly associated with patient survival by Cox analysis. (B) KM curve shows that the high ratio of *FAP* ^+^ CAF group based on the best cutoff grouping had shorter overall survival; barplot shows that the *FAP* ^+^ CAF proportion was significantly higher in different liver cancer samples compared to adjacent liver (AL); boxplot shows the differences in *FAP* ^+^ CAF proportion among different clinical characteristic groups. *, *P* < 0.05; **, *P* < 0.01; ***, *P* < 0.001; ****, *P* < 0.0001. (C) UMAP shows prognostic-associated cells and their tissue-type origins identified by the Scissor algorithm. (D) KM curves show poorer survival of patients with a high proportion of Scissor^+^ cells in the single-cell discovery cohort. (E) Bar plot showing the proportion of Scissor^+^ cells in different tissue types. (F) Pie plot showing the percentage of different fibroblast subtypes in Scissor-related cells. (G) KM curves showing the high *FAP* ^+^ CAF score group usually predicted worse overall survival in the five liver cancer bulk transcriptome cohorts. (H) Fibroblast differentiation trajectories showing the distribution of cell type, sample type, Scissor type and state.

Tumor cells often alter the surrounding microenvironment, frequently accompanied by changes in cellular ratios. The association between cell proportion and patient risk was initially evaluated in the discovery single-cell cohort. We found that Fb_03_FAP (*HR* = 1.7, Cox *P* = 0.00049) and Fb_08_GPM6B (*HR* = 1.4, Cox *P* = 0.00063) were significantly associated with worse overall survival (OS) of patients **(Figure 2A)**. However, Fb_08 was excluded from further analysis due to its low cell count (n = 88). Upon clinical indicator assessment, high infiltration of *FAP* ^+^ CAFs was associated with worse OS (Log-rank *P* < 0.05), lymph node metastasis (Wilcoxon test *P* < 0.01), distal metastasis (Wilcoxon test *P* < 0.0001), and high stage (Wilcoxon test *P* < 0.0001) in liver cancer patients, but there was no significant correlation with viral infection** (Figure 2B)**.

To integrate and analyze bulk RNA-seq data with single-cell data, we used the Scissor algorithm developed by Sun *et al.* to study the association of cells with patient survival 32, 51. Based on TCGA bulk RNA-seq and survival data, we identified Scissor^+^ cells from fibroblasts that were associated with shorter OS **(Figure 2C)**, the finding that was further validated in single-cell discovery cohort **(Figure 2D)**. By assessing the proportion of cells in different sample types, Scissor^+^ cells were higher in ICC compared to HCC samples** (Figure 2E)**. It is noteworthy that *FAP* ^+^ CAFs constitute a significant component of Scissor^+^ cells, specifically accounting for a remarkable 72% in the cell subtype proportion analysis **(Figure 2F)**. Like *FAP* ^+^ CAFs, the Scissor^+^ cells also exhibited high expression of genes related to blood vessel development (*SEPRINE1*, *VEGFA*, *CXCL8*, *THBS1*), and which were more inclined to be highly expressed in ICC **(Figure S3A & S3B)**. Furthermore, in five independent bulk RNA-seq cohorts, a high *FAP* ^+^ CAF signature score was found to predict worse OS of patients** (Figure 2G)**. These results indicate that *FAP* ^+^ CAF significantly affects the clinical outcome of patients and is strongly associated with tumor metastasis and higher aggressiveness.

To explore the origin of *FAP*^+^ CAFs, we conducted a pseudotime trajectory analysis to model fibroblast differentiation. We hypothesized that AL-enriched fibroblasts were used as the starting point of differentiation, and Fb_03 and Fb_02 were found in the end of the two differentiation trajectories **(Figure 2H)**. Fb_02 is primarily derived from SMCs or myofibroblasts (Fb_01), whereas Fb_03 potentially derived from hepatic stellate cells (HSCs) (Fb_04) based on its downstream position in Fb_04 differentiation and similar gene expression profiles **(Figure 2H & 1G)**. Activation of HSCs is thought to have an important role in the development of HCC and is closely related to the differentiation of matrix-associated CAFs 52.

### Different spatial distribution of *FAP* ^+^ CAFs in HCC and ICC

**Figure 3:**
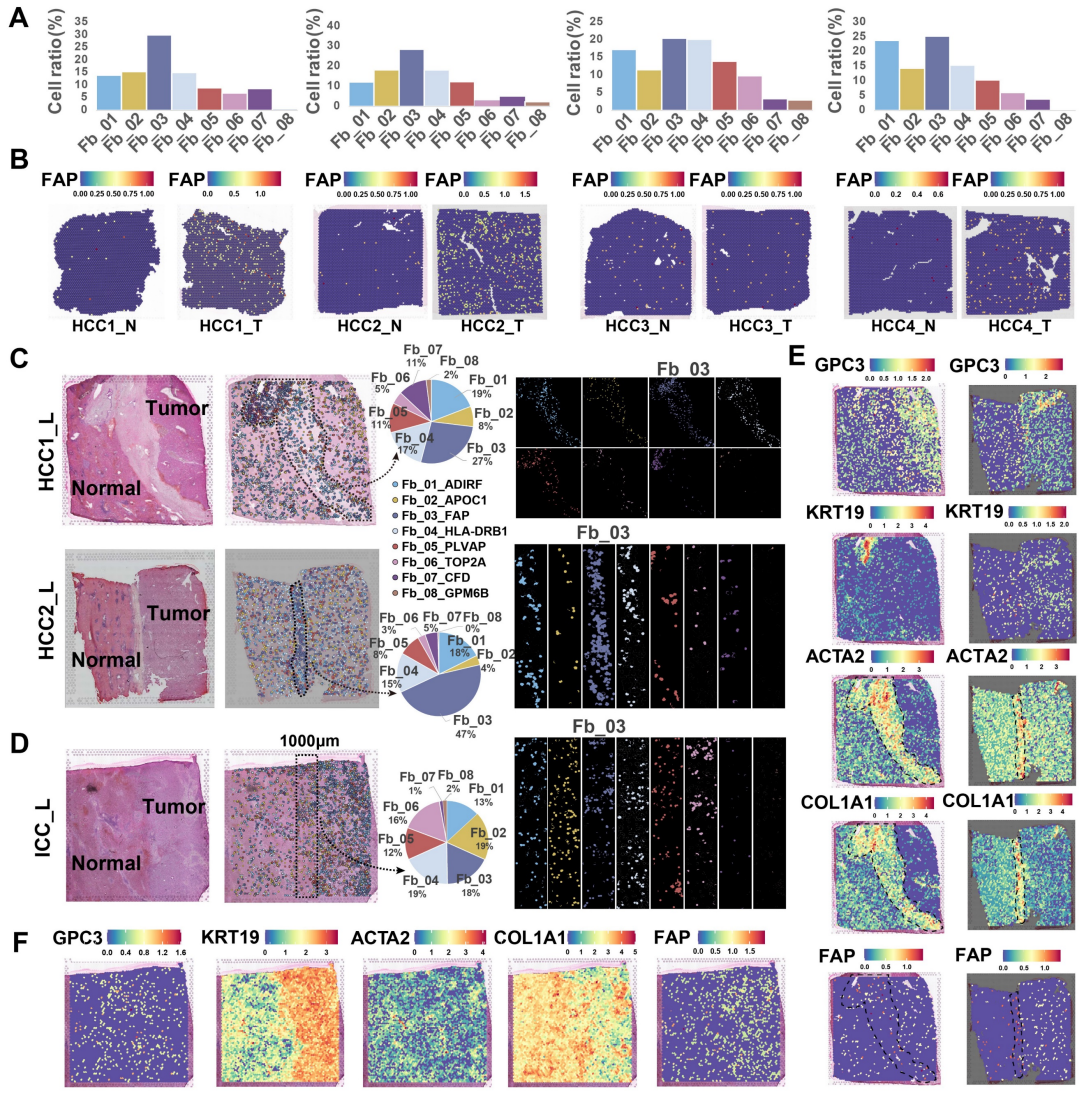
**Spatial distribution of *FAP* ^+^ CAF.** (A) Bar plot shows the proportion of fibroblast subtypes that were deconvoluted by CellTrek onto hepatocellular carcinoma (HCC) spatial transcriptome sections. (B) Spatial feature plot showing the expression of *FAP* in tumor and paracancerous samples from four HCC patients. (C and D) Distribution of fibroblast subtypes in HCC and intrahepatic cholangiocarcinoma (ICC) border slides based on CellTrek deconvolution. The pie charts show the relative proportion of each cell subtype in the border region. (E and F) Spatial feature plot showing the expression of selected genes in HCC and ICC border samples.

The spatial distribution of cells within TME is often closely related to their functional attributes. To gain a deeper understanding of how *FAP*^+^ CAFs contribute to cancer progression, we conducted a ST RNA-seq analysis to map the spatial patterns of *FAP* ^+^ CAFs. First, the proportion of different fibroblast subtypes in the HCC samples was evaluated, and *FAP* ^+^ CAF subtype exhibited the highest cell percentage compared to other subtypes (HCC1_T: 30.05%, HCC2_T: 28.61%, HCC3_T: 20.54%, HCC4_T: 25.38%) **(Figure 3A)**. Furthermore, *FAP* expression was observed to be higher in tumor samples compared to AL **(Figure 3B)**, which was consistent with the IHC results **(Figure 1N)**, and further supported by IF analysis **(Figure S4)**. Next, by evaluating the distribution of fibroblasts in tumor border samples, we found that *FAP* ^+^ CAFs were more likely to be enriched at the tumor border in HCC samples while diffusely scattered in ICC **(Figure 3C-3F)**.

To identify cell types closely linked to fibroblasts, we analyzed five bulk RNA-seq datasets (n = 1303). Through deconvolution, we estimated the proportions of main cell types in each sample and found a strongest correlation between macrophages and fibroblasts across multiple datasets (TCGA, *R* = 0.49, *P* < 2.2e-16; GSE54236, *R* = 0.24, *P* = 0.0023; GSE76427, *R* = 0.27, *P* = 0.00044; GSE14520, *R* = 0.24, *P* = 4e-07; GSE116174, *R* = 0.34, *P* = 0.0058)** (Figure S5A)**. Furthermore, spatial correlation and co-localization analysis using ST data revealed a strong association and spatial proximity between macrophages and fibroblasts **(Figure S5B & 5C)**. Overall, *FAP* ^+^ CAFs are enriched in tumor samples, particularly at HCC borders, and exhibit significant interaction potential with macrophages.

### Spatial co-localization between *FAP* ^+^ CAFs and TAMs

**Figure 4:**
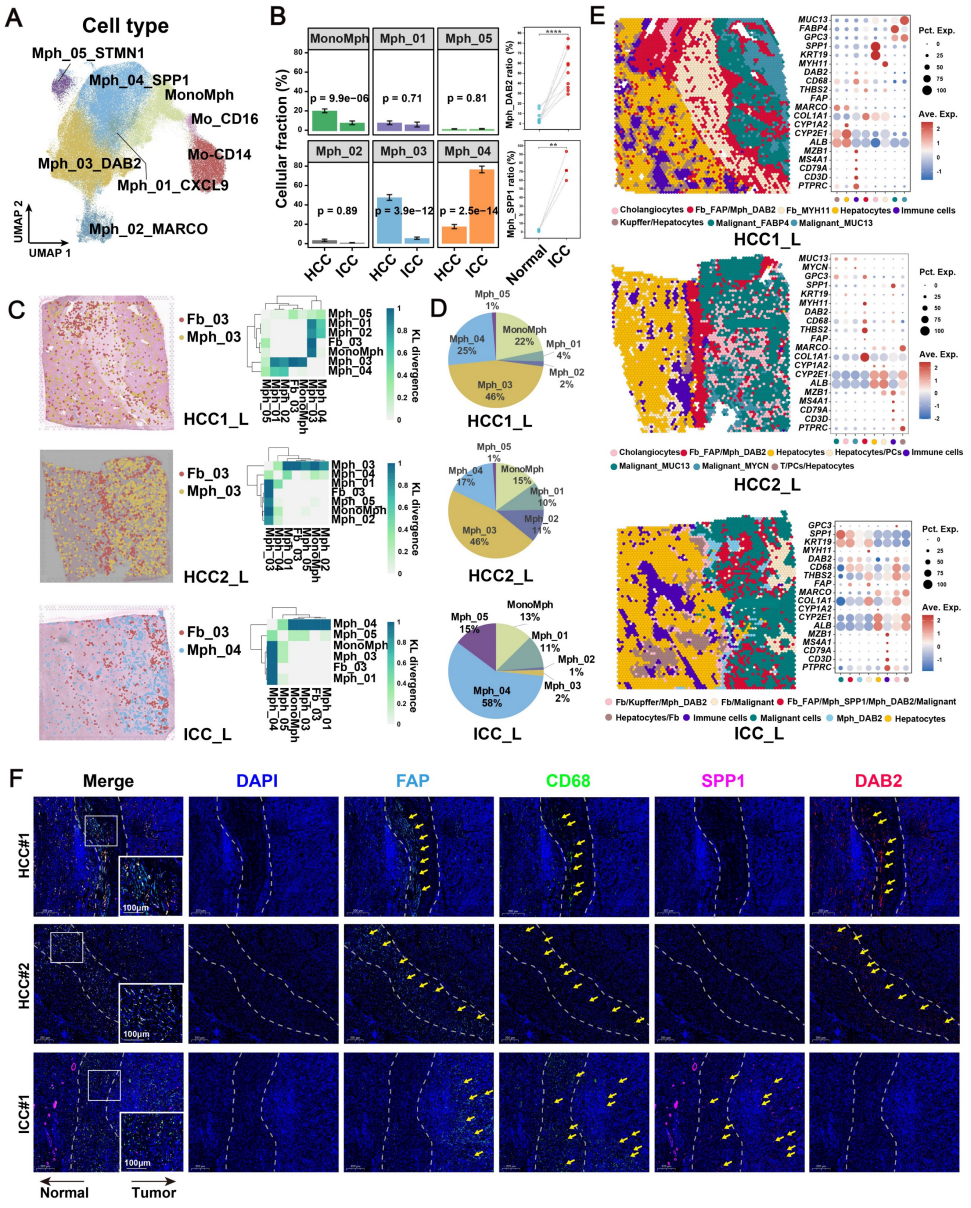
**Spatial co-localization of TAM with *FAP* ^+^ CAF.** (A) UMAP shows the distribution of monocyte/macrophage subtypes. (B) Bar plot shows the relative proportions of macrophage subtypes in HCC and ICC samples (left); paired dot plot shows the relative proportions of *DAB2* ^+^ / *SPP1* ^+^ TAMs in tumor and adjacent liver paired samples (right). *, *P* < 0.05; **, *P* < 0.01; ***, *P* < 0.001; ****, *P* < 0.0001. (C) Distribution of *DAB2* ^+^ TAMs and *FAP* ^+^ CAF in HCC boundary slides, and *SPP1* ^+^ TAM and *FAP* ^+^ CAF in ICC boundary slides based on CellTrek deconvolution (left); heatmap shows the Kullback-Leibler (KL) divergence of *FAP* ^+^ CAF with different macrophage subtypes in ST slides, with the higher KL divergence representing the greater degree of co-localization of the two cell types (right). (D) Pie plots showing the relative proportions of different macrophage subtypes in the ST slides. (E) Unbiased clustering of ST spots and definition of cell types of each cluster (left); dot plot showing the expression of select marker genes of each cluster (right). (F) Multi-plex immunofluorescence images showing the aggregation of *FAP* ^+^ CAF with *DAB2* ^+^ TAM at the tumor border in HCC and *FAP* ^+^ CAF with *SPP1* ^+^ TAM at the tumor border and core in ICC. The scale bar is 200 μm and 100 μm.

To identify the major macrophage subtypes that communicate with *FAP*^+^ CAFs, we performed subtype annotation of macrophages and identified a total of eight major cell subtypes. These included classical CD14 and nonclassical CD16-expressing monocytes in blood 53 and six tissue macrophage subtypes **(Figure 4A, Table S4)**. The macrophage clusters exhibit a greater proclivity for specific sample types and a tendency to exhibit bias, which may be related to the high heterogeneity of macrophages 46, 54** (Figure S6A)**. Subsequently, we conducted an investigation into the enrichment preference and infiltration proportions of cellular subtypes in different sample types. Our findings revealed that mononuclear-like macrophages (MonoMph, *FCN1*^+^) and hepatic Kupffer-like macrophages (Mph_02_MARCO, *MARCO*^+^) 55 were predominant in AL, with Mph_02_MARCO being the most abundant **(Figure S6B)**. Macrophages marked by high CXCL9 expression (Mph_01_CXCL9) are often associated with a more favorable prognosis in tumor patients 56. These macrophages are more enriched in CHC **(Figure S6B)**. Conversely, *SPP1*^+^ macrophages (Mph_04_SPP1), reported with pro-tumorigenic roles, showed a stronger preference for enrichment in CHC, ICC, and SLC samples **(Figure S6B)**. Notably, Mph_03_DAB2 were specifically enriched in HCC. RNA velocity analysis indicated that Mph_03_DAB2 was more likely to be differentiated from resident Kupffer-like cells, while Mph_04_SPP1 was likely to originate from blood-infiltrating MonoMph or resident Kupffer-like cells **(Figure S6C)**.

A further functional comparison revealed that Mph_01_CXCL9 scored highest in M1 phenotype, whereas Mph_03_DAB2 and Mph_04_SPP1 leaned toward the M2 phenotype (**Figure S6D**). Compared with Mph_04_SPP1, Mph_03_DAB2 exhibiting higher levels of *FOLR2*, *SLC40A1*, and *IGF1*, which are genes specifically expressed by some reported TAMs that known to play a role in promoting tumor progression 57-59 **(Figure S6E)**. By utilizing cell-specific marker scores and survival analysis, we found that patients with high Mph_03_DAB2 or Mph_04_SPP1 scores had shorter overall survival (OS)** (Figure S6F)**. Interestingly, although both tumor-promoting TAMs were increased in tumor compared to the AL samples, Mph_03_DAB2 was higher in HCC, but Mph_04_SPP1 was higher in ICC, as we verified in single-cell cohorts, bulk cohorts, and IF staining **(Figure 4B, Figure S6G & S6H)**.

*FAP*^+^ CAFs and different types of macrophages were mapped onto spatial tissue images by CellTrek's ST and scRNA-seq co-embedding and random forest model prediction. The highest spatial co-localization ability of Fb_03_FAP and Mph_03_DAB2 was observed in HCC samples, while in ICC samples, Fb_03_FAP had the highest spatial co-localization with Mph_04_SPP1** (Figure 4C)**. In terms of cell ratio, Mph_03_DAB2 exhibited the highest proportion in HCC slides, while Mph_04_SPP1 in ICC slides **(Figure 4D)**. This finding was consistent with the results of scRNA-seq **(Figure 4B)**. Further spatial cellular annotation revealed that *FAP* ^+^ CAFs and TAMs were co-localized in the HCC border and scattered in ICC tissue, and the co-localized spot always enriched on the ECM-related terms **(Figure 4E, Figure S7A-S7C)**. This observation aligns with the findings of IF experiments **(Figure 4F)**. The results indicate that *FAP* ^+^ CAF may contribute to immune cell exclusion 60, 61 as T/B cells were often absent from the tumor core **(Figure 4E, Figure S8)**. In conclusion, our findings indicate that *DAB2* ^+^ and *SPP1* ^+^ macrophages are the dominant TAMs in liver cancer. Notably, *DAB2* ^+^ TAMs enriched in HCC, exhibiting significant spatial co-localization with *FAP* ^+^ CAF in tumor border and may participate in shaping the TME.

### TAMs enhance *FAP* ^+^ CAFs function through cell communication

**Figure 5:**
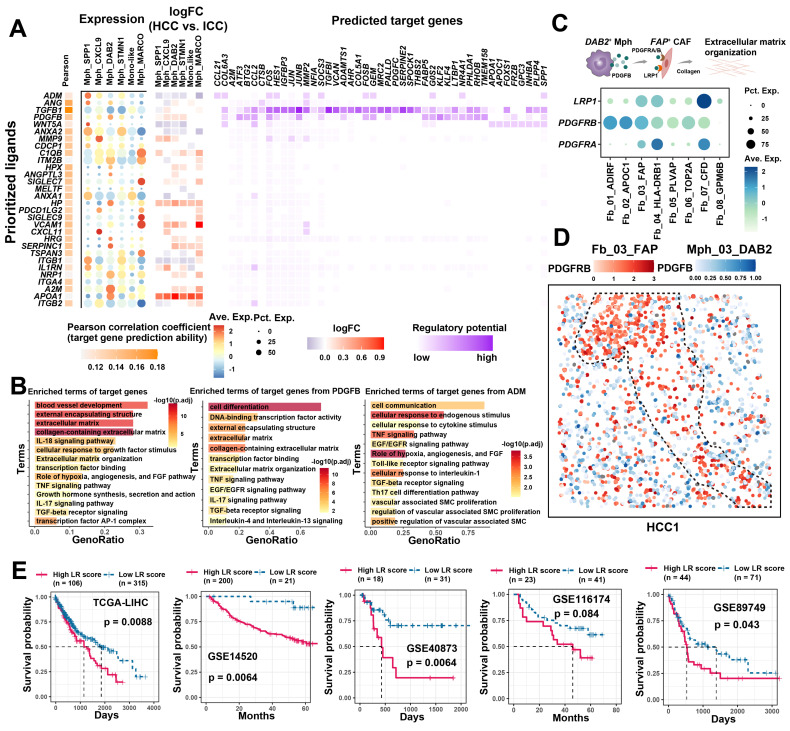
** Cellular communication between TAM and *FAP* ^+^ CAF.** (A) The combined heatmap shows the results after NicheNet analysis of TAM and *FAP* ^+^ CAF. The first part of the combined figure shows the Pearson coefficient of the macrophage ligand, and the high coefficient suggests that the ligand has a high ability to regulate the *FAP* ^+^ CAF target genes, the second part shows the expression of the ligand in different subtypes of macrophage, and the third part shows the comparison of the expression of the ligand in HCC and ICC, and the fourth part shows the regulated potential of the target genes. (B) Bar plot shows GO biology terms enriched for all targeted genes, *PDGFB* target genes, and ADM target genes. (C) Dot plot showing the expression of *PDGFB* receptors *LRP1*, *PDGFRB* and *PDGFRA* in fibroblasts. (D) Spatial dot plot showing the spatial expression of *PDGFB* in *DAB2* ^+^ TAM and *PDGFRB* in *FAP* ^+^ CAF. (E) KM curves showing the association of quantified ligand-receptor score (LRscore) with patient OS in five independent bulk RNA-seq cohorts.

The significant association and spatial co-localization characteristics of *FAP* ^+^ CAFs with TAM prompted us to further explore their potential cellular communication patterns. First, we used *FAP*^+^ CAFs as a receiver to investigate the functional regulation affected by TAM **(Figure 5A)**. Most of the ligand and receptor genes are usually associated with worse patient survival, implying that the interaction between TAMs and *FAP*^+^ CAFs might be tumor-promoting **(Figure S9A)**. Concurrently, we found that target genes in *FAP* ^+^ CAF were mainly involved in blood vessel development, extracellular matrix, collagen-containing extracellular matrix, and cellular response to growth factor stimulus **(Figure 5B)**. Among the ligands from TAM, *TGFB1*, which has the highest regulatory activity on the target genes of *FAP* ^+^ CAF, has been reported to be a key driver gene for fibroblast activation and is widely expressed in macrophages **(Figure 5A)**. Additionally, some macrophage subtype-specific genes were identified that may play an important role in the function regulation of CAF, such as *ADM* is highly expressed in Mph_SPP1, and its target genes are involved in pathways related to hypoxia, angiogenesis and vascular-associated smooth muscle cell proliferation regulation **(Figure 5A & 5B)**. The *ADM* has been reported to promote the proliferation of fibroblasts and reduce their apoptosis rate 62. Meanwhile, *PDGFB* was found to be highly expressed in Mph_DAB2, and its target genes were mainly enriched in the terms of cell differentiation, external encapsulating structure, and extracellular matrix **(Figure 5A & 5B)**. We examined the expression of the corresponding receptors for *PDGFB*, of which *PDGFRB*, *LRP1* and *PDGFRA* were all highly expressed in *FAP* ^+^ CAFs. Further spatial expression analysis showed that *PDGFB* was highly spatially proximal to the expression of these receptors and enriched at the HCC boundary **(Figure 5C & 5D, Figure S9B)**. These results suggest that TAM could potentially shape a tumor-promoting TME by interacting with *FAP* ^+^ CAFs.

Furthermore, we also examined the signaling of *FAP*^+^ CAFs to other cells **(Figure S9C)**. In HCC, *FAP*^+^ CAFs sent high weight of signaling to macrophages, while sent more signals to tumor cells and endothelial cells in ICC, which is similar to previous study 63. CellChat signaling pathway analysis revealed that COLAGEN, MIF, CXCL and VEGF signals were significantly enriched in the outgoing signals of *FAP* ^+^ CAFs **(Figure S9D)**. Next, we examined the effects of *FAP*^+^ CAFs on macrophages in HCC, and some ligands associated with cell migration and macrophage differentiation were identified, such as *BMP4* and *CSF1* **(Figure S9E & S9F)**, which may play key roles in macrophage recruitment and pro-M2 polarization 64, 65. Specifically, ligand-receptor interactions between *FAP*^+^ CAFs and tumor cells and endothelial cells in ICC were investigated. Growth factors were highlighted due to their significant enrichment and role as key molecules in CAF regulation of tumor/endothelial cells **(Figure 5B)** 66. We found VEGFB presented high communication weight and ICC expression specificity, which were associated with shorter OS in ICC patients **(Figure S10A-S10C)**. Moreover, a significant positive correlation was observed between VEGFB and receptors RAMP1 and CALCRL of the *SPP1*^+^ TAMs ligand ADM **(Figure S10D)**. *SPP1*^+^ TAMs may promote *FAP* ^+^ CAFs proliferation through ADM-(CALCRL/RAMP1), and *FAP* ^+^ CAFs may modulate endothelial and tumor cell function and growth by VEGFB **(Figure S10E & S10F)** 67. In summary, *FAP*^+^ CAFs play a pivotal role in the TME, contributing to tumor barrier formation, angiogenesis, and direct tumor cell regulation, albeit with possible preferences for specific cancer subtypes. TAMs are implicated in enhancing the downstream functions of *FAP^+^* CAFs through cellular communication.

### Cell communication quantification and drug screening

Cellular communication largely influences patient prognosis and anti-tumor immunity 68. Utilizing the ssGSEA method, we quantified the signal strength from TAM to *FAP*^+^ CAFs, termed the LRscore, to investigate its association with patient prognosis in a bulk RNA-seq cohort. Across five independent liver cancer cohorts, a higher LRscore consistently predicted a shorter OS for patients **(Figure 5E)**. These findings suggest that disrupting the cellular interaction between TAM and *FAP*^+^ CAFs could potentially serve as a valuable clinical adjuvant strategy. To explore potential targeted drugs that might benefit patients with a high LRscore, we first calculated Spearman's correlation between predicted IC50 values of drugs included in the Genomics of Drug Sensitivity in Cancer (GDSC) database and the LRscore using the previously reported method based on ridge regression models 43. Among the predicted drugs, Syk inhibitors such as Entospletinib (*R* = -0.44, *P* = 4.87E-19) and PRT062607 (*R* = -0.31, *P* = 1.91E-09), as well as Dasatinib (*R* = -0.39, *P* = 7.84E-15) and the PI3K inhibitor AMG-319 (*R* = -0.28, *P* = 6.11E-08), appeared to be more effective in patients with a high LRscore. However, sorafenib, a first-line drug for HCC, showed a positive correlation (R = 0.41, P = 2.73E-16), suggesting it may not be as efficacious in high LRscore patients. Furthermore, we searched for small molecule drugs that are not commonly used in clinical settings but could potentially block this communication. Utilizing the open-source computational tool sc2MeNetDrug 44, we identified small molecule drugs like curcumin, amiloride, and propentofylline as potential cell-communication blocking agents. Curcumin, known for its anti-inflammatory, antioxidant, and antitumor properties, has been reported to be beneficial in the treatment of various cancers 69. Amiloride, a clinically used Na^+^/H^+^ antagonist, inhibits macropinocytosis and enhances the sensitivity of HCC cells to sorafenib-induced iron enrichment 70. These findings offer promising avenues for further exploration in targeting cellular communication to improve patient outcomes.

### Pan-cancer and clinical evaluation of *FAP*^+^ CAFs and *DAB2*^+^ TAMs

**Figure 6:**
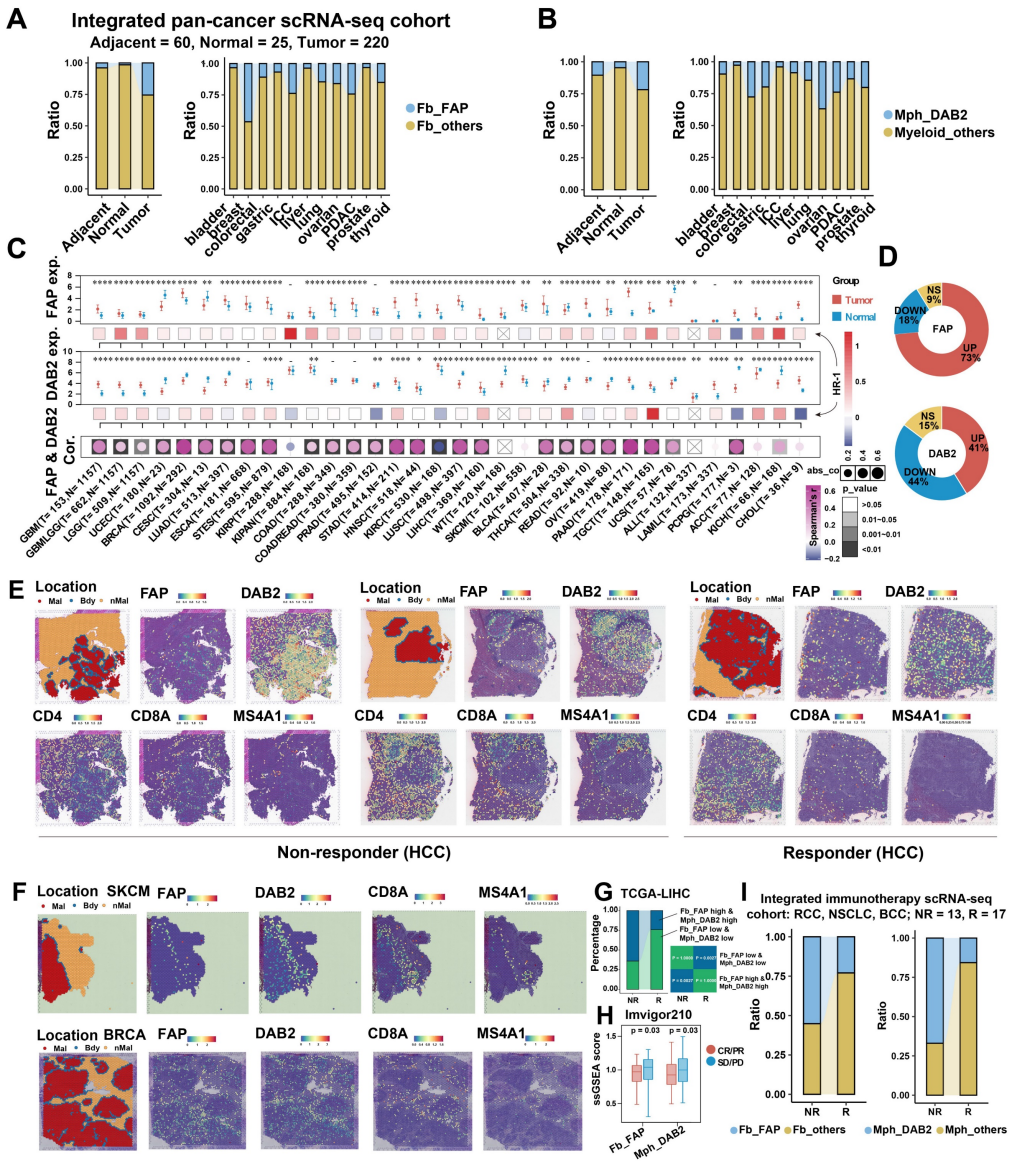
** Pan-cancer analysis of *FAP* ^+^ CAF and *DAB2* ^+^ TAM.** (A and B) Stacking plot showing the proportion of* FAP* ^+^ CAF and *DAB2* ^+^ TAM in different sample types. (C) Gene expression analysis of *FAP* and *DAB2* shows their expression of tumor and normal samples in different cancers, the association between gene expression and patient survival, and the correlation between *FAP* and *DAB2* expression. (D) Circular plots showing the proportion of cancer types with different *FAP* and *DAB2* gene expression patterns, UP indicates the proportion of cancer types in which the gene is significantly up-regulated in cancer, DOWN indicates the proportion of cancer types in which the gene is significantly down-regulated in cancer, and NS indicates the proportion of cancer types in which there is no significant difference in the expression of the gene in cancer and paracancer. (E) Spatial feature plot showing the expression of selected genes in HCC immunotherapy ST slides. (F) Spatial feature plot showing the expression of selected genes in skin cutaneous melanoma (SKCM) and breast invasive carcinoma (BRCA) ST slides. (G) Stacking plot shows the proportion of TCGA-LIHC samples in TIDE-predicted immunotherapy-responsive (R) and non-responsive (NR) samples based on the *FAP* and *DAB2* expression groupings; quad plot shows the significance assessment of the consistency of the four grouping clusters based on the submap method. (H) Box plot comparing the difference between *FAP* ^+^ CAF and *DAB2* ^+^ TAM scores in the response and non-response groups in the immunotherapy cohort Imvigor210. (I) Stacking plot showing the proportion of *FAP* ^+^ CAF and *DAB2* ^+^ TAM in the response and non-response groups in our integrated pan-cancer immunotherapy scRNA-seq cohort.

The findings indicate that *DAB2*^+^ / *SPP1*^+^ TAMs are the dominant TAMs in liver cancer, and *SPP1*^+^ TAMs have been reported to be involved in the pro-tumor microenvironment in several studies 15, 56, 71-73. We questioned whether *DAB2*^+^ TAMs also have a non-negligible role, at least in HCC, where *DAB2*^+^ TAMs abundantly infiltrated and has the highest spatial co-localization with *FAP*^+^ CAFs **(Figure S6B and Figure 4D)**. The interaction between *FAP*^+^ CAFs and *DAB2*^+^ TAMs may be important for the formation of the capsule at the tumor border. To assess the potential significance of *FAP*^+^ CAFs and *DAB2*^+^ TAMs in pan-cancer, it was first necessary to clarify the infiltration abundance of these two cell types in different tumor samples. By analyzing scRNA-seq data from 11 organs, we observed a progressive increase in the proportions of *FAP* ^+^ CAFs and *DAB2* ^+^ TAMs in normal (n = 25), adjacent (n = 60), and tumor (n = 220) samples **(Figure 6A & 6B, Figure S12A & S12B)**. Due to the absence of survival data in the single-cell datasets, we explored the impact of these cell types on the survival of pan-cancer patients using TCGA bulk RNA-seq data. We first evaluated the association of *FAP* and *DAB2* gene expression with the *FAP*^+^ CAF and *DAB2*^+^ TAM ratios calculated by deconvolution in TCGA-LIHC. High *FAP* and *DAB2* group had the highest *FAP*^+^ CAF and *DAB2*^+^ TAM ratios, suggesting that the *FAP* and *DAB2* gene expression can be used for subgroup assessment **(Figure S12C)**. Subsequently, based on gene expression and Cox risk analysis, we found that *FAP* expression was significantly up-regulated in the majority (73%) of cancers (Wilcoxon test, *P* < 0.05), and *DAB2* expression was significantly up-regulated in nearly half of cancers (41%), that high expression of both genes usually predicted poorer OS of the patients **(Figure 6C & 6D, Figure S12D-S12F)**. This suggests that the expression of *FAP* and *DAB2* genes is valuable in indicating the prognosis of tumor patients.

The interaction of TAMs and CAFs in tumor immune barrier (TIB) is associated with the efficacy of immunotherapy by limiting immune infiltration **(Figure S8)**. We therefore investigated the spatial distribution of *FAP*^+^ CAFs and *DAB2*^+^ TAMs in relation to immunotherapy response. In HCC ST slides from immunotherapy-non-responsive patients, *FAP* and *DAB2* were found to be concentrated at the tumor border, creating a barrier that effectively blocked the infiltration of T/B cells. However, in responsive patients, no such enrichment was observed, facilitating efficient T/B cell infiltration **(Figure 6E)**. This trend was also noticeable in various other tumor types **(Figure 6F, Figure S13)**. Significantly, high levels of *FAP*^+^ CAFs and *DAB2*^+^ TAMs were correlated with non-responsiveness to immunotherapy in patients from TCGA, Imvigor210, and integrated single-cell immunotherapy cohorts **(Figure 6G-6I)**. This suggests that the interaction between *FAP*^+^ CAFs and *DAB2*^+^ TAMs could potentially serve as immunotherapy response predictors.

**Figure 7:**
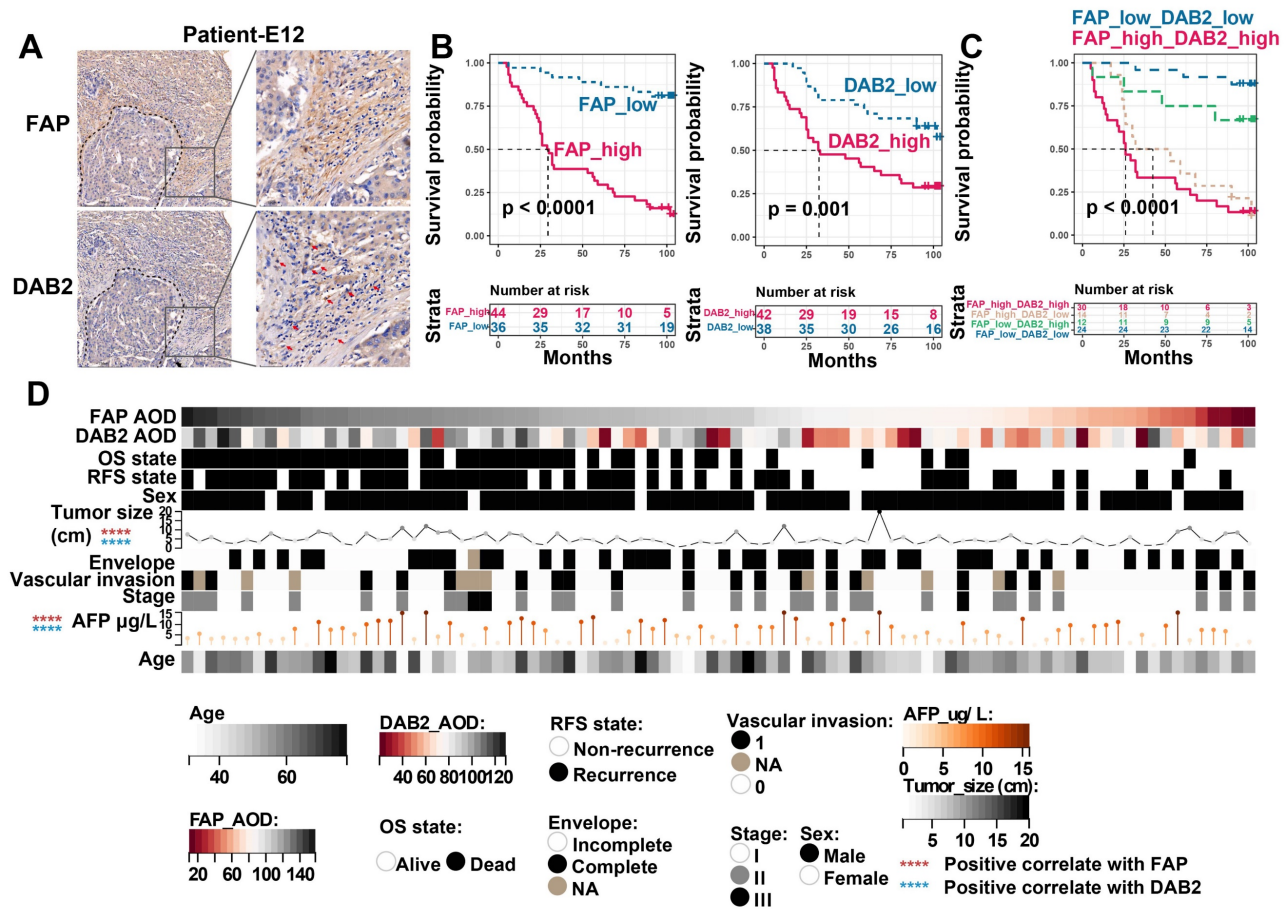
** Immunohistochemical evaluation of FAP and DAB2.** (A) Immunohistochemical staining image shows *FAP* ^+^ CAF and *DAB2* ^+^ TAM around the tumor core. (B) KM curves show the high FAP or DAB2 average optical density (AOD) group with shorter OS. (C) KM curves show the high FAP and DAB2 AOD group had shortest overall survival. (D) Heatmap showing the distribution of clinical indicators in the sample sorted by FAP AOD.

Finally, to evaluate the clinical significance of *FAP* and *DAB2*, we further included 90 HCC tumor boundary samples for IHC staining, in which *FAP* ^+^ CAFs and *DAB2* ^+^ TAMs enriched around the tumor core **(Figure 7A)**. By grouping the patients based on the average optical density (AOD, calculated as integrated optical density divided by area) of FAP or DAB2 staining, we found that patients with elevated levels of FAP (Log-rank *P* < 0.0001) or DAB2 (Log-rank *P* = 0.001) exhibited a reduced OS. Notably, combining both markers (Log-rank *P* < 0.0001) provided an enhanced discriminatory performance **(Figure 7B & 7C)**. Additionally, the staining intensity of FAP and DAB2 also exhibited a significant positive correlation with tumor size and patient serum AFP concentration (Spearman correlation analysis, *P* < 0.0001), suggesting their potential as indicators of tumor progression in clinical practice **(Figure 7D)**.

## Discussion

A growing body of data indicates the potential of immune checkpoint inhibitors in the treatment of liver cancer, especially the single-agent anti-PD-1 immune checkpoint inhibitors have shown promising efficacy in early trials 74, 75. However, majority of patients failing to respond to immunotherapy 76, 77, and the mechanisms underlying the failure to respond remain poorly understood. Tumor heterogeneity, which directly impacts therapeutic targets and shapes the TME by defining transcriptomic and phenotypic profiles, is a significant contributor to immunotherapy inefficacy 78. The growth of solid tumors heavily relies on a remodeled “stroma” comprising CAFs and ECM, which play pivotal roles in shaping the immunosuppressive TME, tumorigenesis, progression, metastasis, and treatment resistance 79. Previous CAF-based single-cell transcriptomic studies in liver cancer have focused on cell type identification, origin, and function, often limited by sample size and fibroblast detection 9, 10, 63, 80-83. It is an urgent need to use scRNA-seq in larger cohorts to study CAFs and their communication with tumor-associated cells. In this study, through multiple cohorts and integrated analysis of scRNA-seq, ST, bulk RNA-seq, and other bioinformatic technologies, we systematically resolved cellular infiltrative alterations in different liver cancer subtypes. Key *FAP*^+^ CAFs were identified, along with extensive communication with *DAB2*^+^ and *SPP1*^+^ TAMs. Notably, the communication between *FAP*^+^ CAFs and *DAB2*^+^ TAMs appear critical in shaping the immune exclusion microenvironment and immunotherapy tolerance in HCC.

Fibroblast heterogeneity endows them with diverse roles in the TME, and single-cell technologies have enabled more precise resolution of this cell population. Several scRNA-seq studies have identified different pan-cancerous CAF subtypes 24, 49, 84, 85, highlighting the importance of matrix-associated CAFs in tumor promotion, characterized by high expression of *COL1A1*, *FAP*, and *POSTN*. These studies are of great significance in resolving the heterogeneity of CAFs and promoting the development of CAF-targeted drugs. In this study, we first identified highest enrichment of fibroblasts in HCC and ICC across multiple liver cancer types. By further subtyping, we identified eight CAF subclusters, *FAP*^+^ CAFs, similar to the previously-reported matrix-associated CAFs, significantly enriched in all liver cancer types, were involved in ECM remodeling and angiogenesis, exhibiting functional heterogeneity across different tumor subtypes. FAP, also known as fibroblast activation protein, is a 97 kDa type II transmembrane serine protease. FAP expression is typically low or undetectable in normal tissues, but is overexpressed in 90% of cancers. To date, FAP has been reported to affect tumor growth through multiple mechanisms, including promotion of proliferation, invasion, angiogenesis, epithelial-mesenchymal transition, stem cell promotion, immunosuppression, and drug resistance 86, 87. Several laboratories around the world have identified a specific subset of FAP^+^ CAF in solid tumors. Importantly, FAP^+^ CAF subpopulation accumulates in cancers with poor prognosis and has been shown to be involved in metastatic spread and cancer immunosuppression 88. Additionally, we found that hypoxia and D-glutamate metabolism were significant features of *FAP* ^+^ CAFs. In fact, hypoxia induces collagen expression and secretion 89-91, and glutamine plays an important role in the promotion of ECM synthesis in fibroblasts by TGF-β 92. Clinical correlation analysis revealed that high infiltration of *FAP*^+^ CAFs correlated with tumor progression and reduced OS.

In recent years, much attention has been paid to tumor boundary, which may help to partially explain the mechanisms of tumor invasive progression and immunotherapy resistance 93, 94. Serving as the predominant CAF subclusters, *FAP*^+^ CAFs were found to be enriched at the tumor border in HCC but diffusely distributed in ICC, which may be related to the formation of a tumor capsule. Visual and pathological examination showed the presence of a capsule around the tumor in 10%-76% of patients with HCC, whereas rarely present in patients with ICC 93. The presence of this fibrous capsule forms a natural physical barrier that prevents immune cells from migrating to the core of the tumor, leading to immune exclusion 22. Actually, we observed that T/B cells were mostly excluded from the border, as described in previous studies 15. Currently, targeted therapeutic options for FAP have been partially reported, including CAR-T therapies, DNA vaccines, antibody targeting FAP, and prodrugs, and a few clinical phase I studies have emphasized their translational potential 95. In recent years, CAR-T therapy have great potential as an emerging immunotherapy strategy. Wang *et al.* developed a second-generation retroviral CAR targeting mouse FAP, and the FAP-CAR-T cells were tested *in vivo* in three different established models, and tumor growth was reduced by 35-50% after treatment 96. The antitumor effects were observed in fully immunoreactive mice but not in immunodeficient mice, suggesting that the depletion of FAP^+^ cells reduce tumor growth in an immune-dependent manner. The combination of FAP-CAR-T cell therapy with immune checkpoint inhibitors may represent an intensive treatment strategy.

Pseudotime trajectory analysis indicated a potential origin of *FAP*^+^ CAFs from HSCs. We found a high similarity in gene expression between *FAP*^+^ CAFs and Fb_04 (similar to HSCs), for example, they both highly express *THBS2* (Figure 1F), which has been reported to be highly expressed in activated HSCs involved in pro-fibrotic processes in the liver 97, which is reminiscent of the myHSC reported by Aveline *et al.* 52. Limiting the activation of HSCs in the TME may help reduce *FAP*^+^ CAFs production and enhance FAP-targeted therapeutic effects 98. *FAP*^+^ CAFs had the strongest spatial co-localization with *DAB2*^+^ TAMs in HCC, and *DAB2*^+^ TAMs had the highest proportion among all macrophages at the tumor border slides. DAB2, also known as Disabled-2, is a clathrin and cargo binding endocytic adaptor protein recognized for its diverse roles in signaling pathways involved in cell differentiation, proliferation, migration, tumor suppression, and other fundamental cellular homeostatic mechanisms 99. Since DAB2 is actively downregulated in a variety of tumor cell lines, it is considered a tumor suppressor 100. However, recent studies have found that *DAB2*^+^ macrophages may promote tumor development. DAB2 is believed to play a role as a regulatory molecule in the process of macrophage phenotypic switching. It is observed to be up-regulated in M2 macrophages and down-regulated in M1 macrophages. High levels of DAB2 expression have been shown to hinder macrophage M1 polarization by inhibiting NF-κB-dependent gene expression 101. *DAB2*^+^ TAMs were also found to localize to the tumor-invasive front and participate in integrin recycling, ECM remodeling, and directed migration 102. Although Liu *et al.* identified *SPP1*^+^ TAMs as the dominant macrophage type in the HCC immune barrier construct 15, *SPP1*^+^ TAMs were predominantly enriched in ICC, in contrast, *DAB2*^+^ TAMs in HCC with higher infiltration **(Figure 4E)**. Their dominance needs to be revisited, especially in a different tumor context. Our findings indicate that *DAB2*^+^ TAMs are predominantly derived from hepatic Kupffer-like cells, whereas *SPP1*^+^ TAMs are more likely to originate from monocyte-like macrophages, suggesting that they may possess disparate functions. Although they may both promote ECM remodeling through TGF-β signaling, PDGFB and ADM were found to exercise different exclusive functions as specific ligands for *DAB2*^+^ TAMs and *SPP1*^+^ TAMs, respectively. It has been shown that PDGFB directly affects ECM remodeling, with reduced ECM deposition and TGF-β signaling in tumors from mice with a platelet-specific deletion of PDGFB 103. Whereas ADM regulates ECM mainly by controlling fibroblast proliferation and apoptosis, it is important to note that the main function of ADM is to regulate angiogenesis and not ECM 62, 104. Therefore, reducing the infiltration of *DAB2*^+^ TAMs or *SPP1*^+^ TAMs at the tumor border and blocking their cellular communication with *FAP*^+^ CAFs may be another way to enhance anti-tumor immunity.

Several Syk and PI3K inhibitors were identified by our research as having the potential to block the communication between TAMs and *FAP*^+^ CAFs. It has been reported that CAFs increase contractile force and matrix production through the activation of the Syk signaling pathway. We hypothesized that the communication between TAMs and *FAP*^+^ CAFs may involve Syk signaling-mediated ECM remodeling 105. Additionally, there is evidence that PI3K/AKT1 signaling is involved in the activation of CAF and the differentiation of other cells to CAF, and the PI3K inhibitor have been shown to effectively reduce CAF activation 106. However, it is important to note that there is a lack of studies analyzing the effects of these inhibitors on tumor immune barrier formation and immunotherapy.

## Conclusions

In conclusion, our study has uncovered distinct fibroblast profiles across various types of liver cancer. Specifically, we have identified *FAP*^+^ CAFs as a conserved cancer-associated fibroblast subtype that is significantly associated with patient survival, albeit with functional and spatial distribution heterogeneity within different forms of liver cancer. TAMs play an important role in reinforcing the function of *FAP*^+^ CAFs, particularly through the interplay between *DAB2* ^+^ TAMs and *FAP* ^+^ CAFs, which contributes significantly to the establishment of an immune barrier. The exploration and development of targeted therapeutic approaches aimed at *DAB2* ^+^ TAMs, *FAP* ^+^ CAFs, or the molecules mediating their communication hold significant promise for enhancing the efficacy of immunotherapy and ultimately improving patient outcomes.

## Supplementary Material

Supplementary figures.

Supplementary tables.

## Abbreviations

CAFs: Cancer-associated fibroblasts
TAMs: Tumor-associated macrophages
HCC: Hepatocellular carcinoma
ICC: Intrahepatic cholangiocarcinoma
scRNA-seq: Single-cell RNA sequencing
ST: Spatial transcriptome
TME: Tumor microenvironment
POSTN: Periostin
FAP: Fibroblast activation protein
DAB2: Disabled-2
iCAF: Inflammatory CAF
myCAF: Myofibroblastic CAF
apCAF: Antigen-presenting CAF
vCAF: Vascular CAF
ECM: Extracellular matrix
AL: Adjacent liver
CHC: Combined hepatocellular and cholangiocarcinoma
SLC: Secondary liver cancer
PB: Peripheral blood
CNCB: China national center for bioinformation
GEO: Gene expression omnibus
THPA: The human protein atlas
EGA: European genome-phenome archive
GSA: Genome sequence archive
SKCM: Skin cutaneous melanoma
BRCA: Breast cancer
LUAD: Lung adenocarcinoma
RCC: Renal cell carcinoma
MB: Medulloblastoma
PDAC: Pancreatic ductal adenocarcinoma
SCC: Squamous cell carcinoma
EPN: Ependymoma
CRC: Colorectal cancer
HNSC: Head and neck squamous cell carcinoma
OV: Ovarian cancer
PRAD: Prostate adenocarcinoma
GIST: Gastrointestinal stromal tumor
RCC: Renal cell carcinoma
NSCLC: Non-small cell lung cancer
BCC: Basal cell carcinoma
FFPE: Formalin-fixed paraffin-embedded
PCA: Principal component analysis
HVGs: Highly variable genes
UMAP: Uniform manifold approximation and projection
OR: Odds ratio
KM: Kaplan-Meier
OS: Overall survival
KLD: Kullback-Leibler divergence
PBS: Phosphate-buffered saline
IC50: 50% inhibitory concentration
SMCs: Smooth muscle cells
HSCs: Hepatic stellate cells
GDSC: Genomics of drug sensitivity in cancer
TIB: Tumor immune barrier
AOD: Average optical density
