# Supplementary material for: DAB2+ macrophages support FAP+ fibroblasts in shaping tumor barrier and inducing poor clinical outcomes in liver cancer
Source: Theranostics. 2024 Aug 12;14(12):4822–43. doi: 10.7150/thno.99046 (PMC11373629; doi:10.7150/thno.99046)
Supplement: Supplementary file 1 — Supplementary figures. [file thnov14p4822s1.pdf]

Figure S1. Fibroblast subtype identification. (A) Dot plot showing marker gene expression of major cell types. (B) UMAP shows the sample type and individual sample distribution of all cells. (C) Stacking plot shows the proportion of major cell types in peripheral blood and five tissue samples. (D) UMAP shows the sample type and individual sample distribution of fibroblasts. (E) Feature plot shows the marker gene expression of selected fibroblast subtypes. (F) Heatmap showing sample preference of fibroblast subtypes, where OR > 1.5 was considered significantly enriched for that cell in that type of sample, and OR < 0.5 was considered significantly not enriched. (G) Volcano plot showing the difference in the proportion of major cell types in ICC (n = 31) versus AL (n = 14). (H) Volcano plot comparing the relative abundance of fibroblast subtypes in ICC versus HCC. (I) Heatmap showing the Ucell enrichment scores of key biological entries in the Fb_03_FAP subtype in different tissue types. (J) Bar plot shows the proportion of *FAP* $^{+}$ CAF from TCGA samples after deconvolution by CIBERSORTx. Paired point plot shows the high proportion of *FAP* $^{+}$ CAF in the paired tumor samples from the single-cell discovery cohort. *, $P < 0.05$; **, $P < 0.01$; ***, $P < 0.001$; ****, $P < 0.0001$.

Figure S2. Functional analysis of fibroblast subtypes. (A) Dot plot showing selected biological terms or pathways significantly enriched for each fibroblast subtype. (B) Heatmap shows the enrichment scores of relevant metabolic pathways for fibroblast subtypes calculated by R package scMetabolism; bar plot shows the overall metabolic score of each fibroblast subtype, with the vertical coordinate being the value after scaling of metabolic score. (C) Heatmap showing the enrichment of 50 key cancer hallmarks in different fibroblast subtypes.

Figure S3. Comparison of Scissor cell-related genes and fibroblast subtypes. (A) Volcano plot showing genes differentially expressed between Scissor $^{+}$ and Scissor $^{-}$ cells. Genes with |logFC| > 2, adj. *P* < 0.05 were considered significantly different. (B) Dot plot showing the expression preference of genes highly expressed in Scissor $^{+}$ or Scissor $^{-}$ cells in HCC and ICC.

Figure S4. Fluorescent expression of FAP in tumor and paracancer. Immunofluorescence images show that FAP is more abundantly expressed in tumor samples compared to paracancer. Viewed by SlideViewer with field of view sizes of 2000 μm and 400 μm.

Figure S5. Correlation and co-localization analysis of fibroblasts with macrophages. (A) Heatmap showing Spearman's correlation of the proportion of fibroblasts infiltrating with other major cell types in five independent bulk transcriptome cohorts based on CIBERSORTx deconvolution imputation. Scatterplots show the correlation between the proportion of fibroblasts infiltrated with macrophages. (B) Heatmap showing the correlation of spatial localization between cells based on R package Cards imputed in HCC and ICC ST slides, high correlation indicates high spatial localization between cells. (C) Cell score ST plot showing the presence of spatial co-localization of imputed fibroblasts and macrophages.

Figure S6. Identification of key TAMs. (A) UMAP shows the distribution of sample type and individual sample in macrophage subtypes. (B) Dot plot shows the expression of

macrophage subtype marker genes in different subtypes (left); heatmap shows the tissue enrichment preference of macrophage subtypes (middle); stacking plot shows the relative proportion of macrophage subtypes in different tissues (right). (C) RNA velocity analysis indicates that Mph_DAB2 and Mph_SPP1 are terminally differentiated cell types. (D) Heatmap showing the Ucell enrichment score of macrophage-associated pathways in different macrophage subtypes. (E) Scatter plot showing the differentially expressed genes of *DAB2* $^{+}$TAM (Mph_03) versus *SPP1* $^{+}$TAM (Mph_04). (F) KM curves show that tumor patients with high Mph_03 or Mph_04 score calculated by ssGSEA had worse overall survival. (G) Bar plot showing the proportions of *DAB2* $^{+}$/ *SPP1* $^{+}$ TAMs after deconvolution by CIBERSORTx based on TCGA samples. (H) Immunofluorescence images confirmed that *DAB2* $^{+}$TAM and *SPP1* $^{+}$TAM were enriched in HCC and ICC tumor samples, respectively. Scale bars are 20 μm and 200 μm.

Figure S7. Gene expression and spot annotation of ST boundary slides. (A) Spatial feature plot showing spatial expression of *FAP* $^{+}$CAF and selected macrophage subtype marker genes. (B) Unbiased clustering of ST spots in HCC3 and HCC4 slides and cell type annotation for each cluster. Dot plots showing the expression of specific marker genes for each cluster in HCC3 and HCC4 slides. (C) Dot plot showing the results of GO enrichment analysis of the co-localized regions of *FAP* $^{+}$CAF and TAM.

Figure S8. Spatial distribution of immune cells. (A and B) Malignant spots (Mal, red), boundary spots (Bdy, blue), and

non-malignant spots (nMal, orange) were annotated on the tissue slides by R package Cottrazm. (C and D) Feature plots showing the expression of selected immune cell-related genes. (E and F) Box plots showing the proportion of immune cells in the annotated regions.

Figure S9. *FAP*$^{+}$ CAF recruit macrophages and promotes M2 polarization. (A) Heatmap showing the prior interaction potential of ligand and receptor from TAM to *FAP*$^{+}$ CAF. The dots represent genes significantly associated with survival of TCGA-LIHC patients (Cox $P < 0.05$), red represents better prognosis ($HR > 1$) and blue color represents worser prognosis ($HR < 1$). (B) Spatial dot plot showing the spatial expression of *PDGFB* in *DAB2*$^{+}$TAM and corresponding receptor genes in *FAP*$^{+}$CAF in HCC1_L and HCC2_L slides. (C) Circos plot shows the weights of signal sent by *FAP*$^{+}$CAF to other cell types in HCC or ICC; heatmap shows the weights of signaling exchange between all cell types. (D) Signal enrichment analysis based on cellular communication presents the strength of efferent signaling enrichment pathways of different cell types in HCC or ICC. (E) Heatmap shows the prior interaction potential of ligand and receptor from *FAP*$^{+}$ CAF to TAM; dot plot shows the expression of ligand and receptor genes in fibroblast subtypes and macrophage subtypes. (F) Dot plot showing the pathways to which *FAP*$^{+}$ CAF ligand genes are significantly enriched.

Figure S10. Cell communication of *FAP*$^{+}$ CAF in ICC. (A) Black heatmap shows significant top ligands and receptors between *FAP*$^{+}$CAF and tumor or endothelial cells; red

heatmap shows growth factor-associated ligands and receptors. (B) UMAP and dot plot showing high expression of *VEGFB* in fibroblasts of ICC samples. (C) KM curve showing that ICC patients with high *VEGFB* expression have shorter OS. (D) Scatter plot showing *VEGFB* is significantly positively correlated with *ADM* receptor *RAMP1* and *CALCRL*. (E) Spatial dot plot showing the spatial expression of *ADM* in *SPP1* $^{+}$TAM and *RAMP1* and *CALCRL* in *FAP* $^{+}$ CAF. (F) Conjecture of communication between *SPP1* $^{+}$ TAM, *FAP* $^{+}$CAF, endothelial cells, and tumor cells in ICC.

Figure S11. Drugs predicted to block TAM-*FAP* $^{+}$ CAF interaction. (A) Dot plot showing the targeted drugs with the high correlation with the LRscore based on TCGA-LIHC samples. A higher positive correlation means that the drug is more sensitive for patients with a low LRscore, and a negative correlation means that the drug is more sensitive for patients with a high LRscore. (B and C) Bar plot showing the small molecule drugs predicted by the R package sc2MeNetDrug to block TAM-*FAP* $^{+}$ CAF communication, where a high negative enrichment score represents a higher likelihood that the drug will work; the network plot shows clustering based on drug structure.

Figure S12. Single-cell and bulk pan-cancer analysis of *FAP* $^{+}$CAF and *DAB2* $^{+}$TAM. (A and B) UMAP show the identification of *FAP* $^{+}$CAF and *DAB2* $^{+}$TAM and associated gene expression from the integrated pan-cancer single-cell cohort. (C) Bar plot showing the proportion of *FAP* $^{+}$CAF and *DAB2* $^{+}$TAM infiltration based on gene *FAP* and *DAB2* expression grouping. (D) KM curves showing that high *FAP*

or *DAB2* gene expression is associated with worse OS in pan-cancer patients. (E) KM curve showing that patients with high *FAP* and *DAB2* gene expression had the shortest OS. (F) KM curves for 39 cancers show that patients with high *FAP* and *DAB2* gene expression usually predict shorter OS. (G and H) UMAP showing identification of *FAP* $^{+}$ CAF and *DAB2* $^{+}$ TAM from an integrated pan-cancer immunotherapy single-cell cohort; dot plot showing selected subtype-specific gene expression.

Figure S13. Spatial distribution of *FAP* and *DAB2* at the boundaries of multiple cancers. Tissue slides were annotated by malignant spots (Mal, red), boundary spots (Bdy, blue), and non-malignant spots (nMal, orange), including lung adenocarcinoma (LUAD, GSM5420751), renal cell carcinoma (RCC, GSM5924036), medulloblastoma (MB, EGAS00001006124), pancreatic ductal adenocarcinoma (PDAC, GSM6505134), squamous cell carcinoma (SCC, V10F24_015_A1, doi.org/10.17632/2bh5fchcv6.1), ependymoma (EPN, GSM5844724), colorectal cancer (CRC, P6, HRA000979), head and neck squamous cell carcinoma (HNSC, GSM5494476), ovarian cancer (OV, Human Ovarian Cancer, 11 mm Capture Area from 10x), prostate adenocarcinoma (PRAD, EGAS00001006124), gastrointestinal stromal tumor (GIST, GSM6177607).

Figure S1.

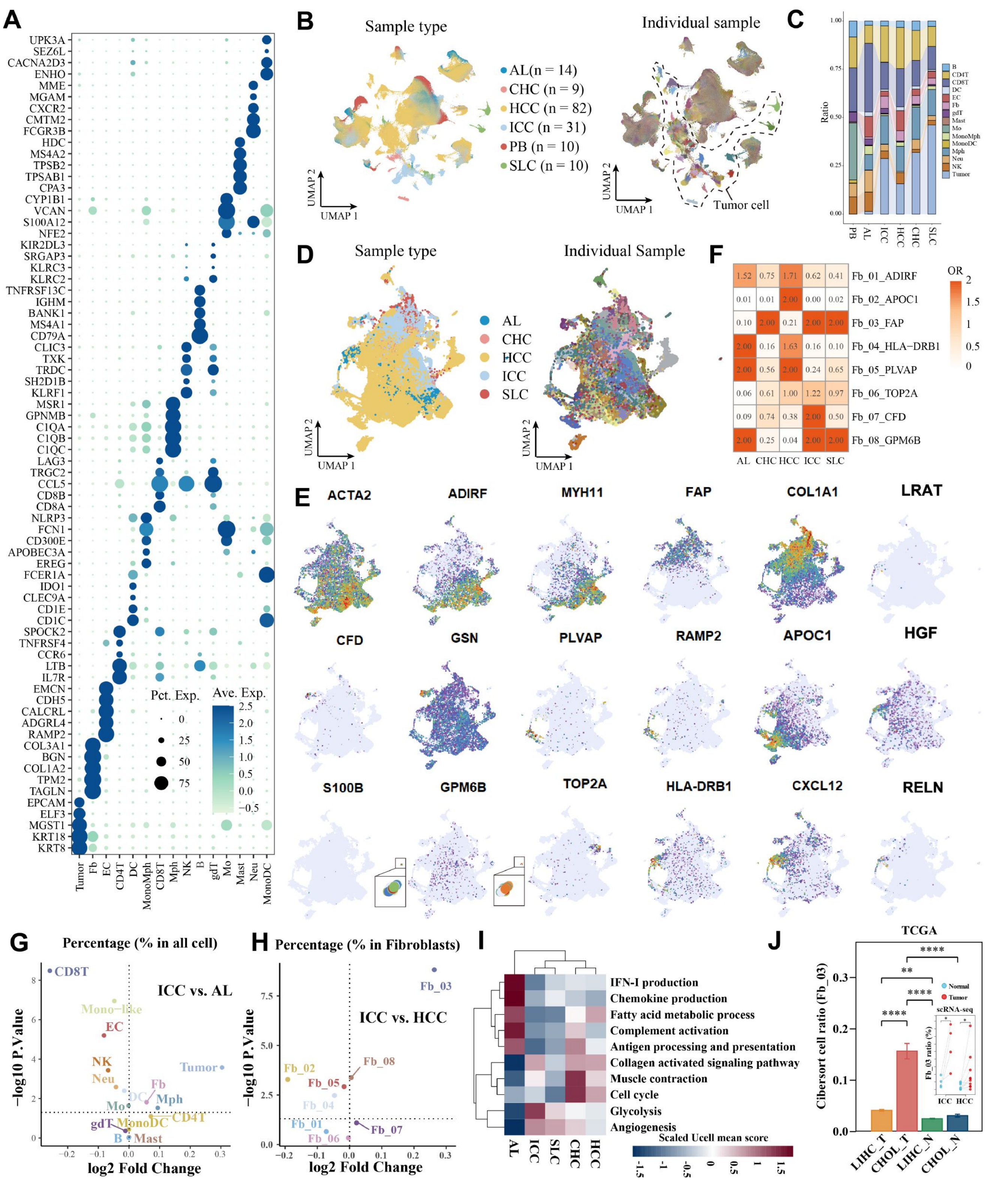

A
B
Sample type
Individual sample
AL(n = 14)
CHC (n = 9)
HCC (n = 82)
ICC (n = 31)
PB (n = 10)
SLC (n = 10)
Tumor cell
UMAP 1
UMAP 2
C
Ratio
D
Sample type
Individual Sample
AL
CHC
HCC
ICC
SLC
E
ACTA2
ADIRF
MYH11
FAP
COL1A1
LRAT
CFD
GSN
PLVAP
RAMP2
APOC1
HGF
S100B
GPM6B
TOP2A
HLA-DRB1
CXCL12
RELN
F
OR
Fb_01_ADIRF
Fb_02_APOC1
Fb_03_FAP
Fb_04_HLA−DRB1
Fb_05_PLVAP
Fb_06_TOP2A
Fb_07_CFD
Fb_08_GPM6B
Pct. Exp.
Ave. Exp.
G
Percentage (% in all cell)
ICC vs. AL
−log10 P.Value
log2 Fold Change
H
Percentage (% in Fibroblasts)
ICC vs. HCC
I
IFN-I production
Chemokine production
Fatty acid metabolic process
Complement activation
Antigen processing and presentation
Collagen activated signaling pathway
Muscle contraction
Cell cycle
Glycolysis
Angiogenesis
Scaled Ucell mean score
J
TCGA
Cibersort cell ratio (Fb_03)
Normal
Tumor
scRNA-seq
LIHC_T
CHOL_T
LIHC_N
CHOL_N

Figure S2.

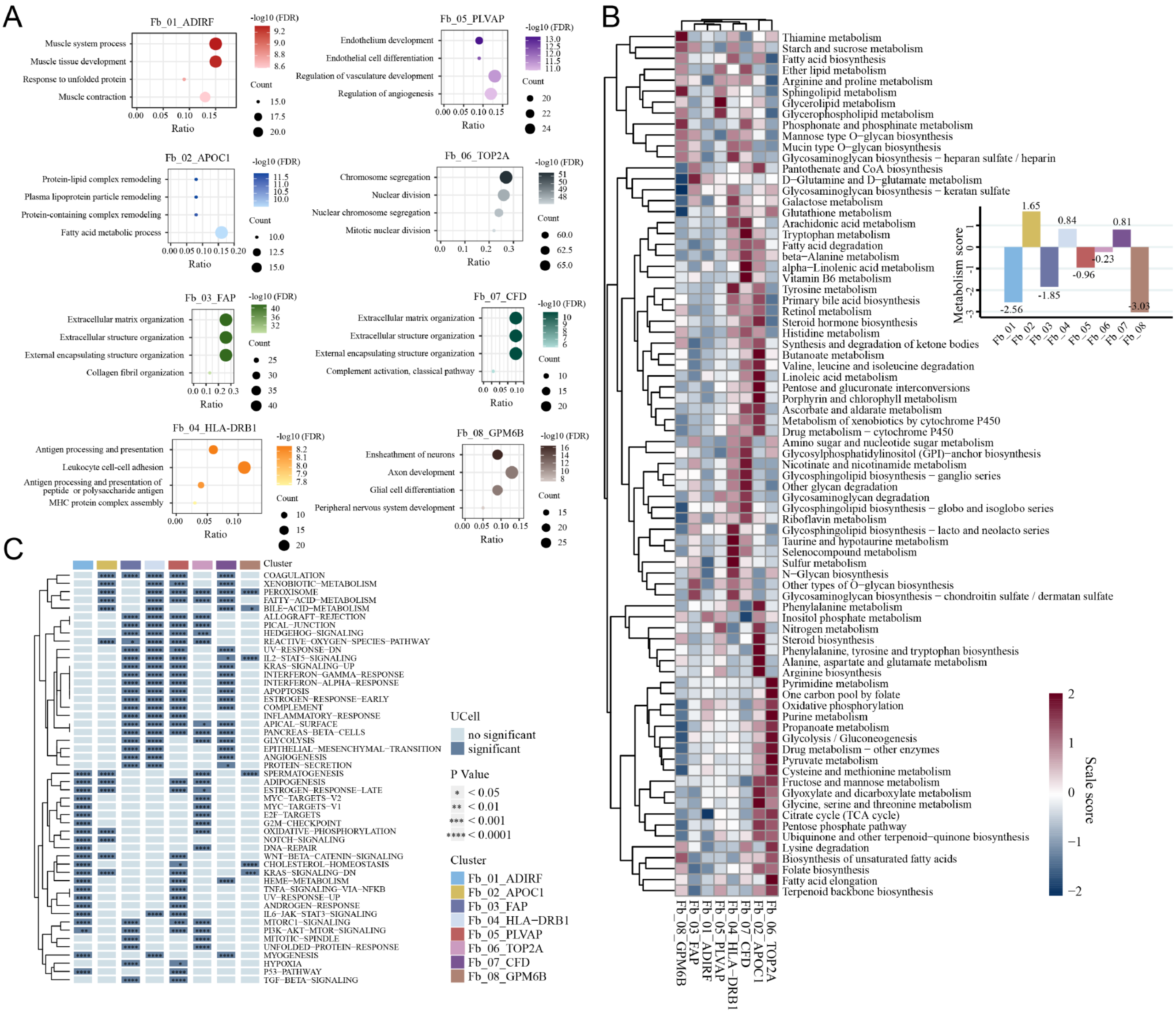

Figure S3.

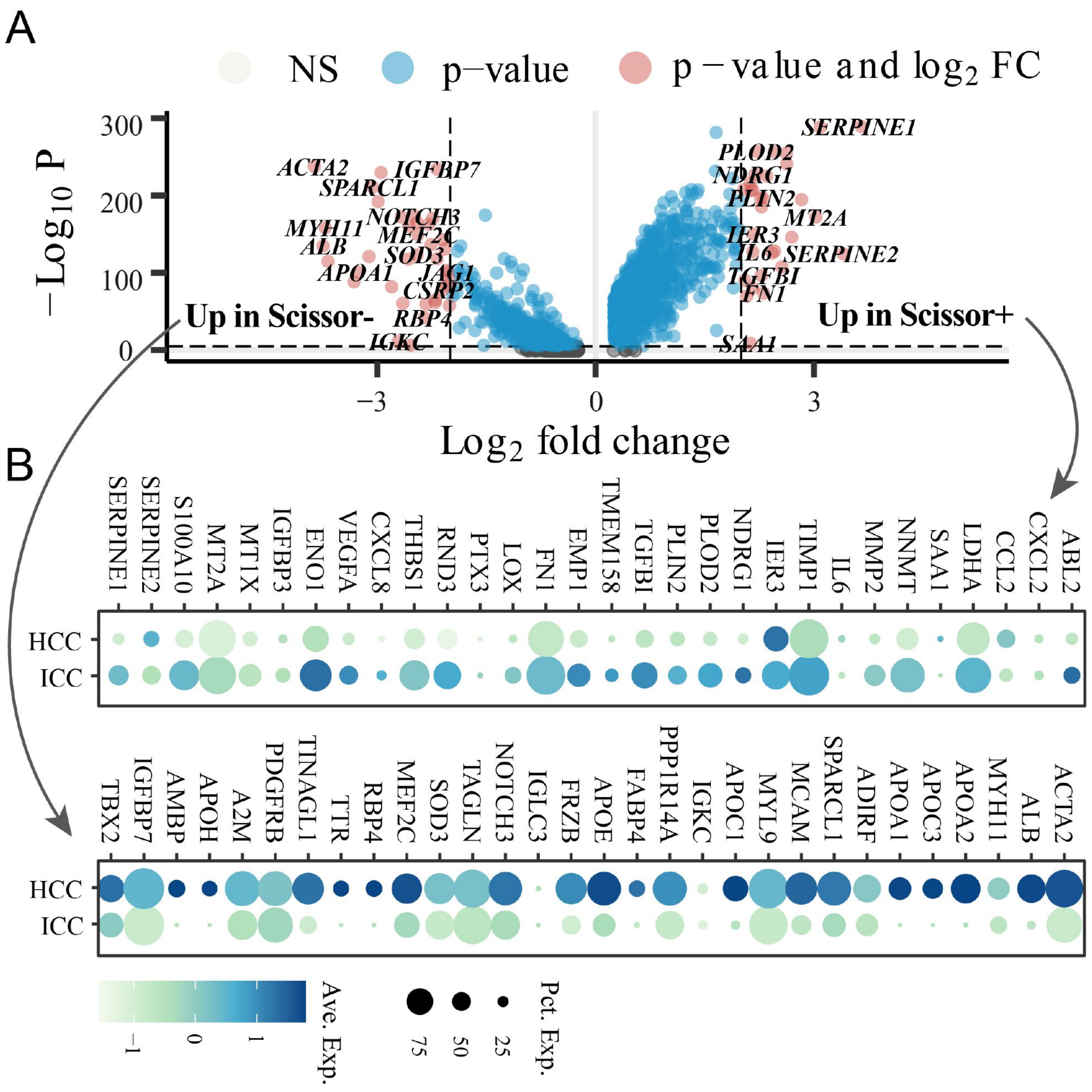

A
NS
p−value
p − value and log2 FC
−Log10 P
Log2 fold change
Up in Scissor-
Up in Scissor+
SERPINE1
PLOD2
NDRG1
PLIN2
MT2A
IER3
IL6
SERPINE2
TGFBI
FN1
SAA1
ACTA2
IGFBP7
SPARCL1
NOTCH3
MYH11
MEF2C
ALB
SOD3
APOA1
JAG1
CSRP2
RBP4
IGKC
B
HCC
ICC
Ave. Exp.
Pct. Exp.

Figure S4.

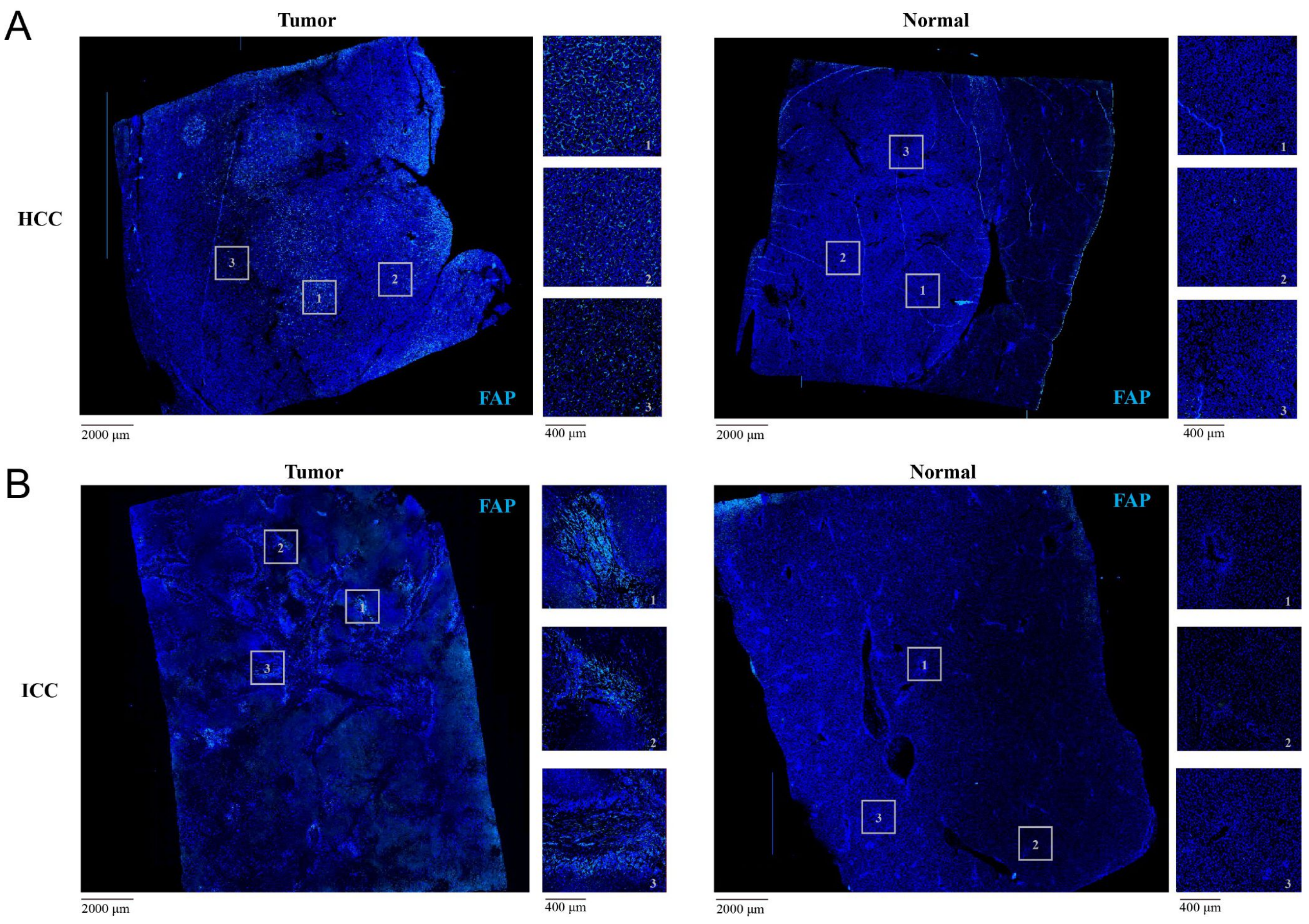
A
Tumor
Normal
HCC
FAP
FAP
2000 µm
400 µm
2000 µm
400 µm
B
Tumor
Normal
ICC
FAP
FAP
2000 µm
400 µm
2000 µm
400 µm

Figure S5.

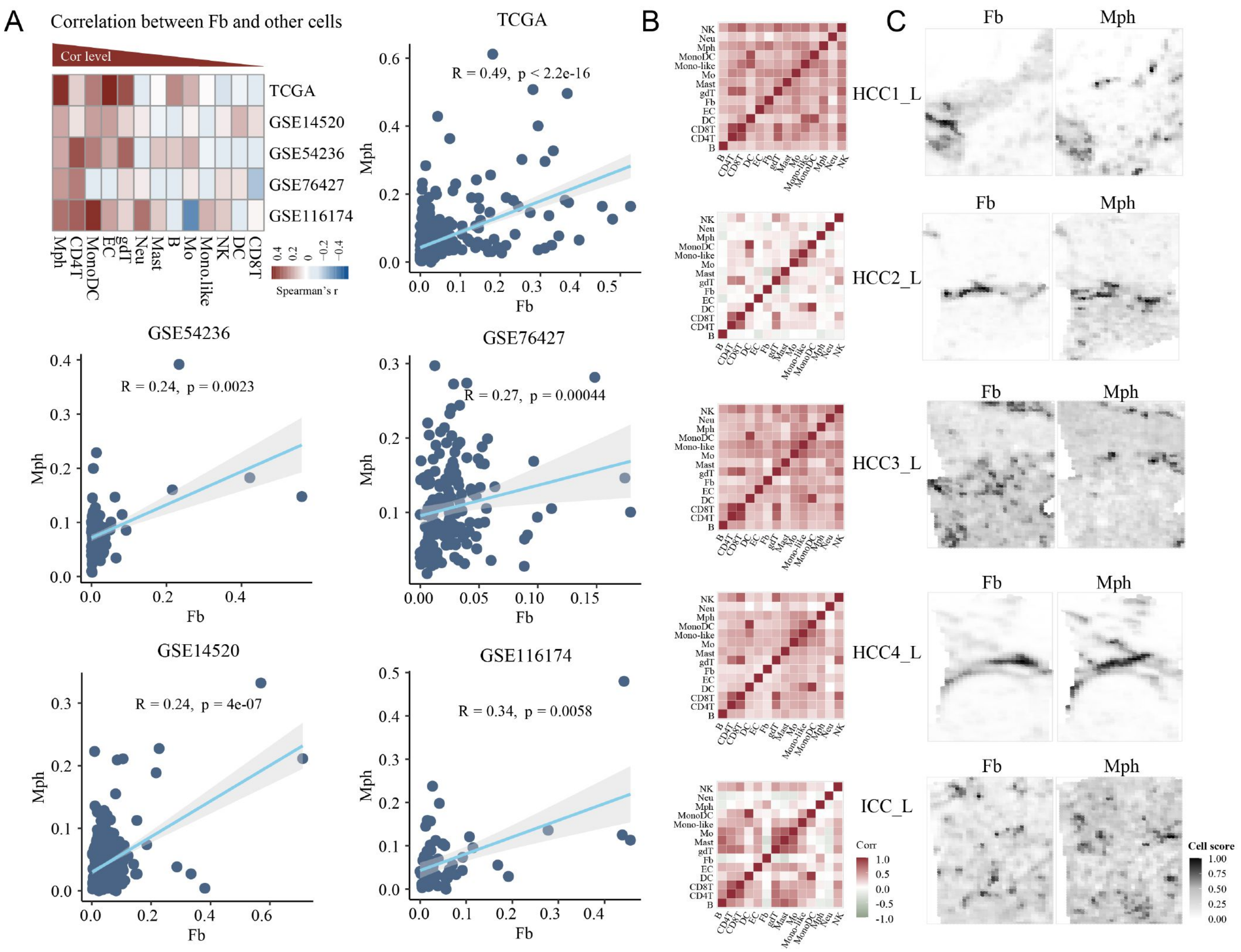
A
Correlation between Fb and other cells
Cor level
TCGA
GSE14520
GSE54236
GSE76427
GSE116174
Mph
CD4T
MonoDC
EC
gdT
Neu
Mast
B
Mo
Mono.like
NK
DC
CD8T
Spearman's r
TCGA
R = 0.49, p < 2.2e-16
GSE54236
R = 0.24, p = 0.0023
GSE76427
R = 0.27, p = 0.00044
GSE14520
R = 0.24, p = 4e-07
GSE116174
R = 0.34, p = 0.0058
Mph
Fb
B
HCC1_L
HCC2_L
HCC3_L
HCC4_L
ICC_L
Corr
C
Fb
Mph
Cell score

Figure S6.

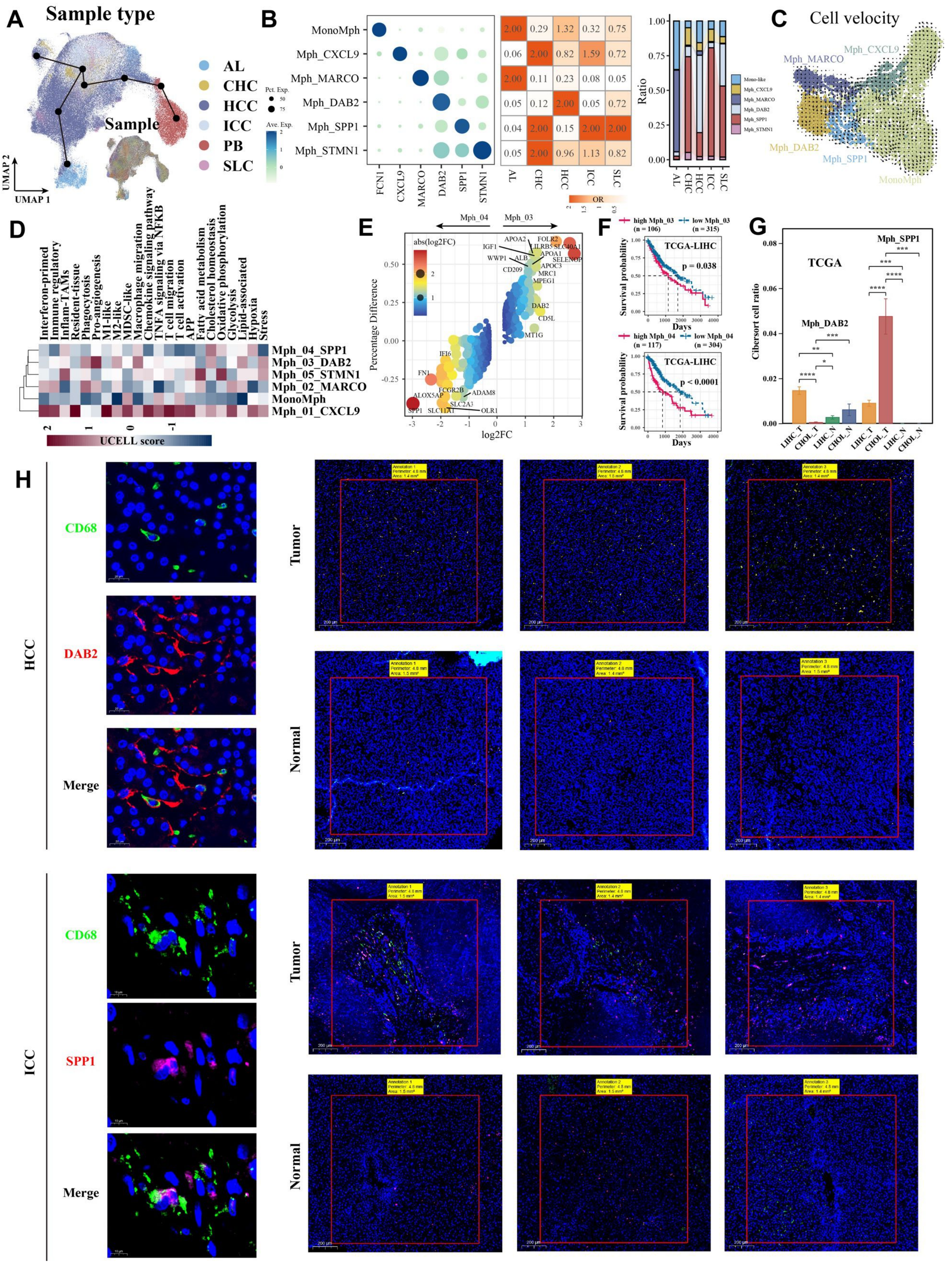
A
Sample type
Sample
AL
CHC
HCC
ICC
PB
SLC
UMAP 1
UMAP 2
B
MonoMph
Mph_CXCL9
Mph_MARCO
Mph_DAB2
Mph_SPP1
Mph_STMN1
FCN1
CXCL9
MARCO
DAB2
SPP1
STMN1
Pct. Exp.
Ave. Exp.
AL
CHC
HCC
ICC
SLC
OR
Ratio
C
Cell velocity
Mph_MARCO
Mph_CXCL9
Mph_DAB2
Mph_SPP1
MonoMph
D
Interferon-primed
Immune regulatory
Inflam-TAMs
Resident-tissue
Phagocytosis
Pro-angiogenesis
M1-like
M2-like
MDSC-like
Macrophage migration
Chemokine signaling pathway
TNFA signaling via NFKB
T cell migration
T cell activation
APP
Fatty acid metabolism
Cholesterol homeostasis
Oxidative phosphorylation
Glycolysis
Lipid-associated
Hypoxia
Stress
Mph_04_SPP1
Mph_03_DAB2
Mph_05_STMN1
Mph_02_MARCO
MonoMph
Mph_01_CXCL9
UCELL score
E
Mph_04
Mph_03
abs(log2FC)
Percentage Difference
log2FC
F
TCGA-LIHC
p = 0.038
p < 0.0001
Survival probability
Days
G
TCGA
Mph_SPP1
Mph_DAB2
Cibersort cell ratio
LIHC_T
CHOL_T
LIHC_N
CHOL_N
H
HCC
CD68
DAB2
Merge
Tumor
Normal
ICC
CD68
SPP1
Merge
Tumor
Normal

Figure S7.

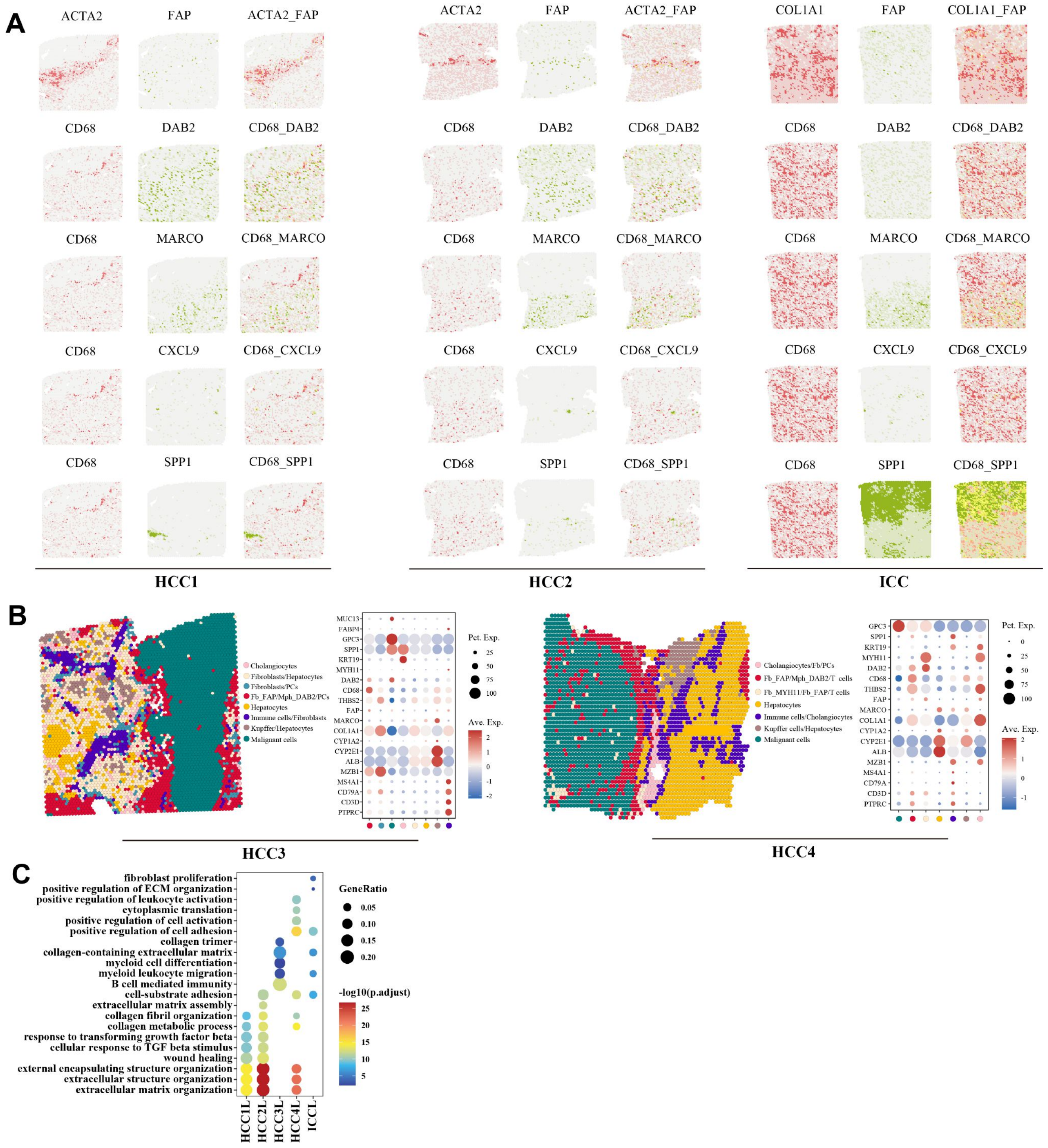
A
ACTA2
FAP
ACTA2_FAP
COL1A1
COL1A1_FAP
CD68
DAB2
CD68_DAB2
MARCO
CD68_MARCO
CXCL9
CD68_CXCL9
SPP1
CD68_SPP1
HCC1
HCC2
ICC
B
Cholangiocytes
Fibroblasts/Hepatocytes
Fibroblasts/PCs
Fb_FAP/Mph_DAB2/PCs
Hepatocytes
Immune cells/Fibroblasts
Kupffer/Hepatocytes
Malignant cells
Pct. Exp.
Ave. Exp.
HCC3
Cholangiocytes/Fb/PCs
Fb_FAP/Mph_DAB2/T cells
Fb_MYH11/Fb_FAP/T cells
Hepatocytes
Immune cells/Cholangiocytes
Kupffer cells/Hepatocytes
Malignant cells
HCC4
C
fibroblast proliferation
positive regulation of ECM organization
positive regulation of leukocyte activation
cytoplasmic translation
positive regulation of cell activation
positive regulation of cell adhesion
collagen trimer
collagen-containing extracellular matrix
myeloid cell differentiation
myeloid leukocyte migration
B cell mediated immunity
cell-substrate adhesion
extracellular matrix assembly
collagen fibril organization
collagen metabolic process
response to transforming growth factor beta
cellular response to TGF beta stimulus
wound healing
external encapsulating structure organization
extracellular structure organization
extracellular matrix organization
HCC1L
HCC2L
HCC3L
HCC4L
ICCL
GeneRatio
0.05
0.10
0.15
0.20
-log10(p.adjust)
25
20
15
10
5

Figure S8.

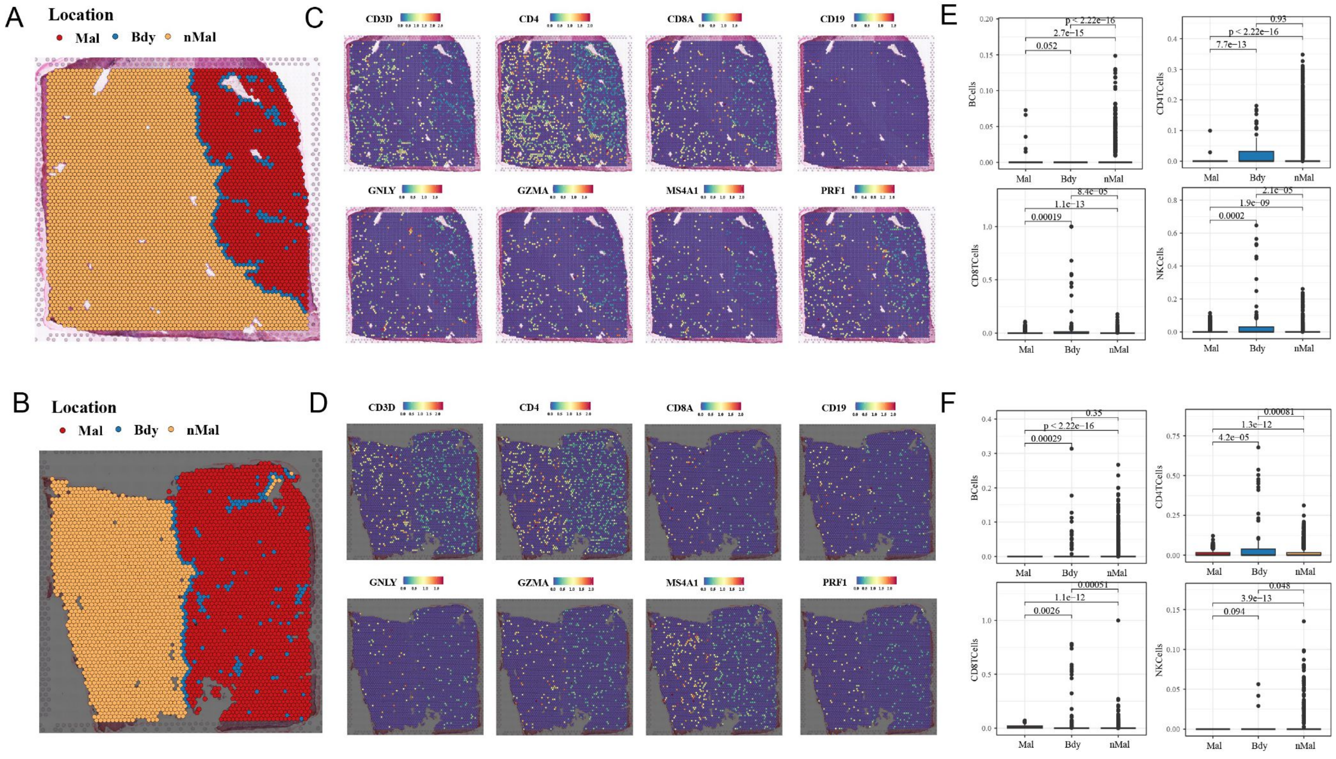

A
Location
Mal
Bdy
nMal
B
Location
Mal
Bdy
nMal
C
CD3D
CD4
CD8A
CD19
GNLY
GZMA
MS4A1
PRF1
D
CD3D
CD4
CD8A
CD19
GNLY
GZMA
MS4A1
PRF1
E
BCells
CD4TCells
CD8TCells
NKCells
Mal
Bdy
nMal
p < 2.22e-16
2.7e-15
0.052
0.93
p < 2.22e-16
7.7e-13
8.4e-05
1.1e-13
0.00019
2.1e-05
1.9e-09
0.0002
F
BCells
CD4TCells
CD8TCells
NKCells
Mal
Bdy
nMal
0.35
p < 2.22e-16
0.00029
0.00081
1.3e-12
4.2e-05
0.00051
1.1e-12
0.0026
0.048
3.9e-13
0.094

Figure S9.

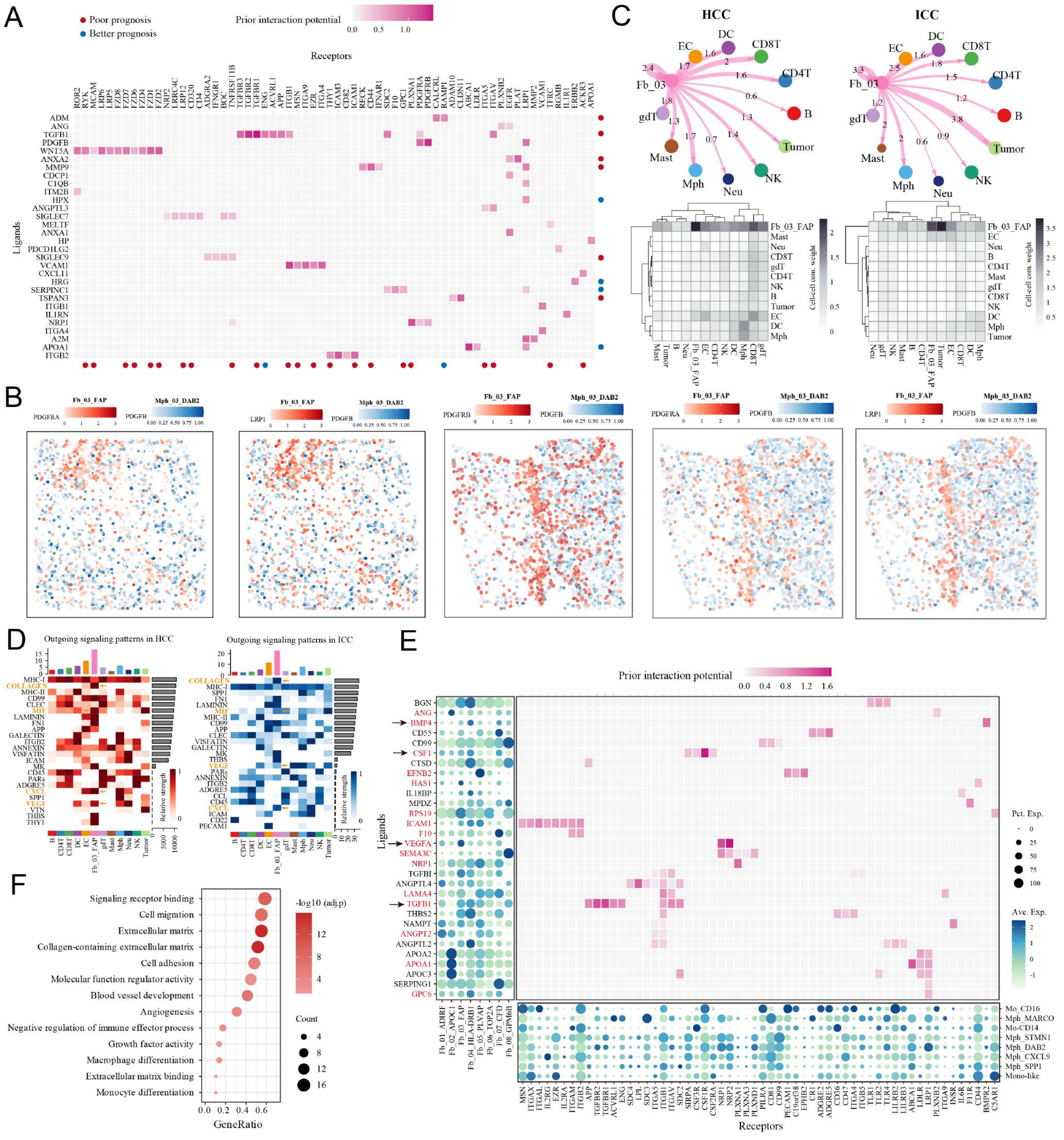
A
Poor prognosis
Better prognosis
Prior interaction potential
Receptors
Ligands
B
Fb_03_FAP
Mph_03_DAB2
C
HCC
ICC
Cell-cell com. weight
D
Outgoing signaling patterns in HCC
Outgoing signaling patterns in ICC
Relative strength
E
Prior interaction potential
Pct. Exp.
Ave. Exp.
Ligands
Receptors
F
Signaling receptor binding
Cell migration
Extracellular matrix
Collagen-containing extracellular matrix
Cell adhesion
Molecular function regulator activity
Blood vessel development
Angiogenesis
Negative regulation of immune effector process
Growth factor activity
Macrophage differentiation
Extracellular matrix binding
Monocyte differentiation
-log10 (adj.p)
Count
GeneRatio

Figure S10.

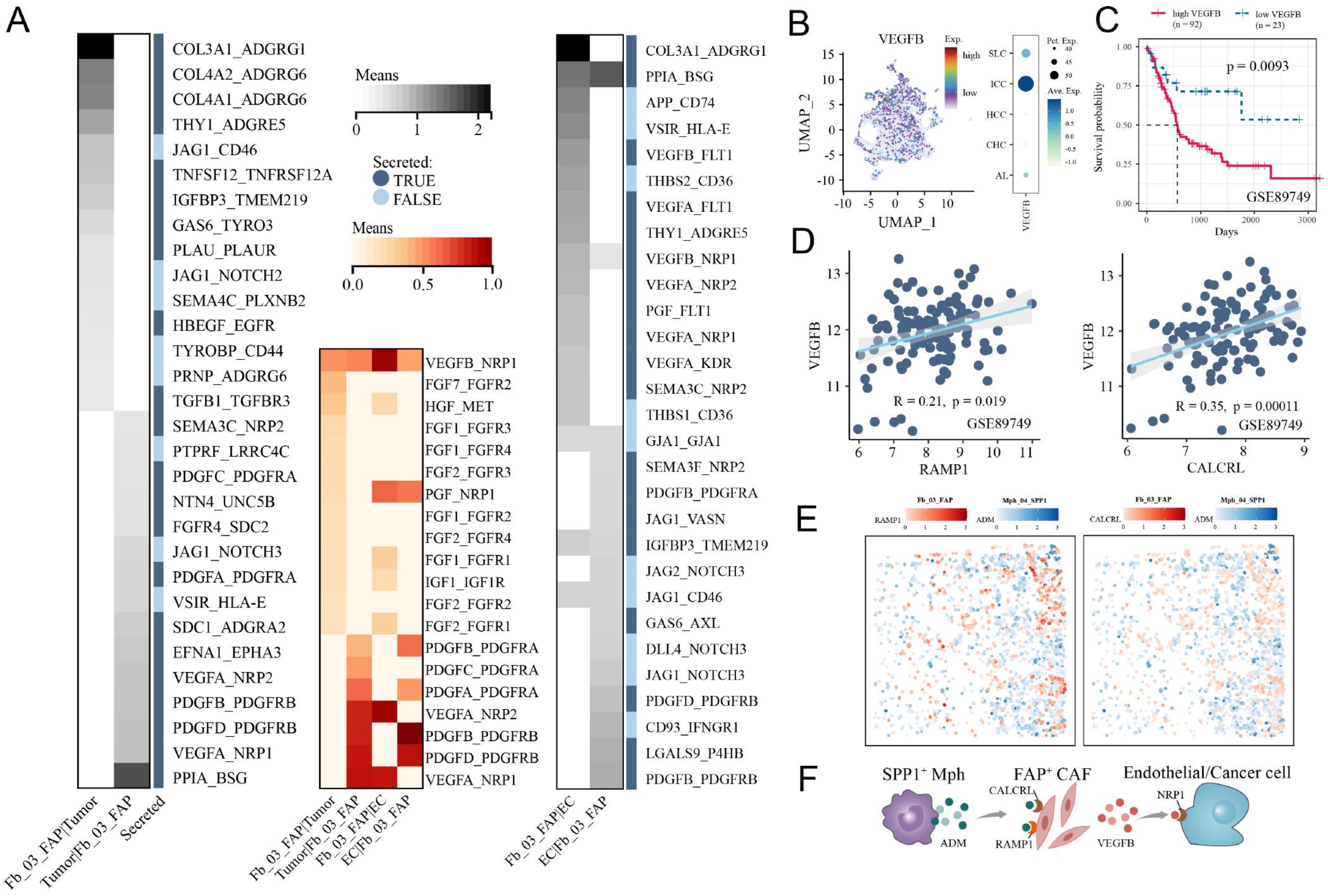

A
COL3A1_ADGRG1
COL4A2_ADGRG6
COL4A1_ADGRG6
THY1_ADGRE5
JAG1_CD46
TNFSF12_TNFRSF12A
IGFBP3_TMEM219
GAS6_TYRO3
PLAU_PLAUR
JAG1_NOTCH2
SEMA4C_PLXNB2
HBEGF_EGFR
TYROBP_CD44
PRNP_ADGRG6
TGFB1_TGFBR3
SEMA3C_NRP2
PTPRF_LRRC4C
PDGFC_PDGFRA
NTN4_UNC5B
FGFR4_SDC2
JAG1_NOTCH3
PDGFA_PDGFRA
VSIR_HLA-E
SDC1_ADGRA2
EFNA1_EPHA3
VEGFA_NRP2
PDGFB_PDGFRB
PDGFD_PDGFRB
VEGFA_NRP1
PPIA_BSG
Fb_03_FAP|Tumor
Tumor|Fb_03_FAP
Secreted
Means
0
1
2
Secreted:
TRUE
FALSE
Means
0.0
0.5
1.0
VEGFB_NRP1
FGF7_FGFR2
HGF_MET
FGF1_FGFR3
FGF1_FGFR4
FGF2_FGFR3
PGF_NRP1
FGF1_FGFR2
FGF2_FGFR4
FGF1_FGFR1
IGF1_IGF1R
FGF2_FGFR2
FGF2_FGFR1
PDGFB_PDGFRA
PDGFC_PDGFRA
PDGFA_PDGFRA
VEGFA_NRP2
PDGFB_PDGFRB
PDGFD_PDGFRB
VEGFA_NRP1
Fb_03_FAP|Tumor
Tumor|Fb_03_FAP
Fb_03_FAP|EC
EC|Fb_03_FAP
COL3A1_ADGRG1
PPIA_BSG
APP_CD74
VSIR_HLA-E
VEGFB_FLT1
THBS2_CD36
VEGFA_FLT1
THY1_ADGRE5
VEGFB_NRP1
VEGFA_NRP2
PGF_FLT1
VEGFA_NRP1
VEGFA_KDR
SEMA3C_NRP2
THBS1_CD36
GJA1_GJA1
SEMA3F_NRP2
PDGFB_PDGFRA
JAG1_VASN
IGFBP3_TMEM219
JAG2_NOTCH3
JAG1_CD46
GAS6_AXL
DLL4_NOTCH3
JAG1_NOTCH3
PDGFD_PDGFRB
CD93_IFNGR1
LGALS9_P4HB
PDGFB_PDGFRB
Fb_03_FAP|EC
EC|Fb_03_FAP
B
VEGFB
Exp.
high
low
UMAP_2
UMAP_1
SLC
ICC
HCC
CHC
AL
Pct. Exp.
Ave. Exp.
VEGFB
C
high VEGFB (n = 92)
low VEGFB (n = 23)
Survival probability
p = 0.0093
GSE89749
Days
D
VEGFB
R = 0.21, p = 0.019
GSE89749
RAMP1
VEGFB
R = 0.35, p = 0.00011
GSE89749
CALCRL
E
Fb_03_FAP
Mph_04_SPP1
RAMP1
ADM
CALCRL
ADM
F
SPP1+ Mph
FAP+ CAF
Endothelial/Cancer cell
CALCRL
ADM
RAMP1
VEGFB
NRP1

Figure S11.

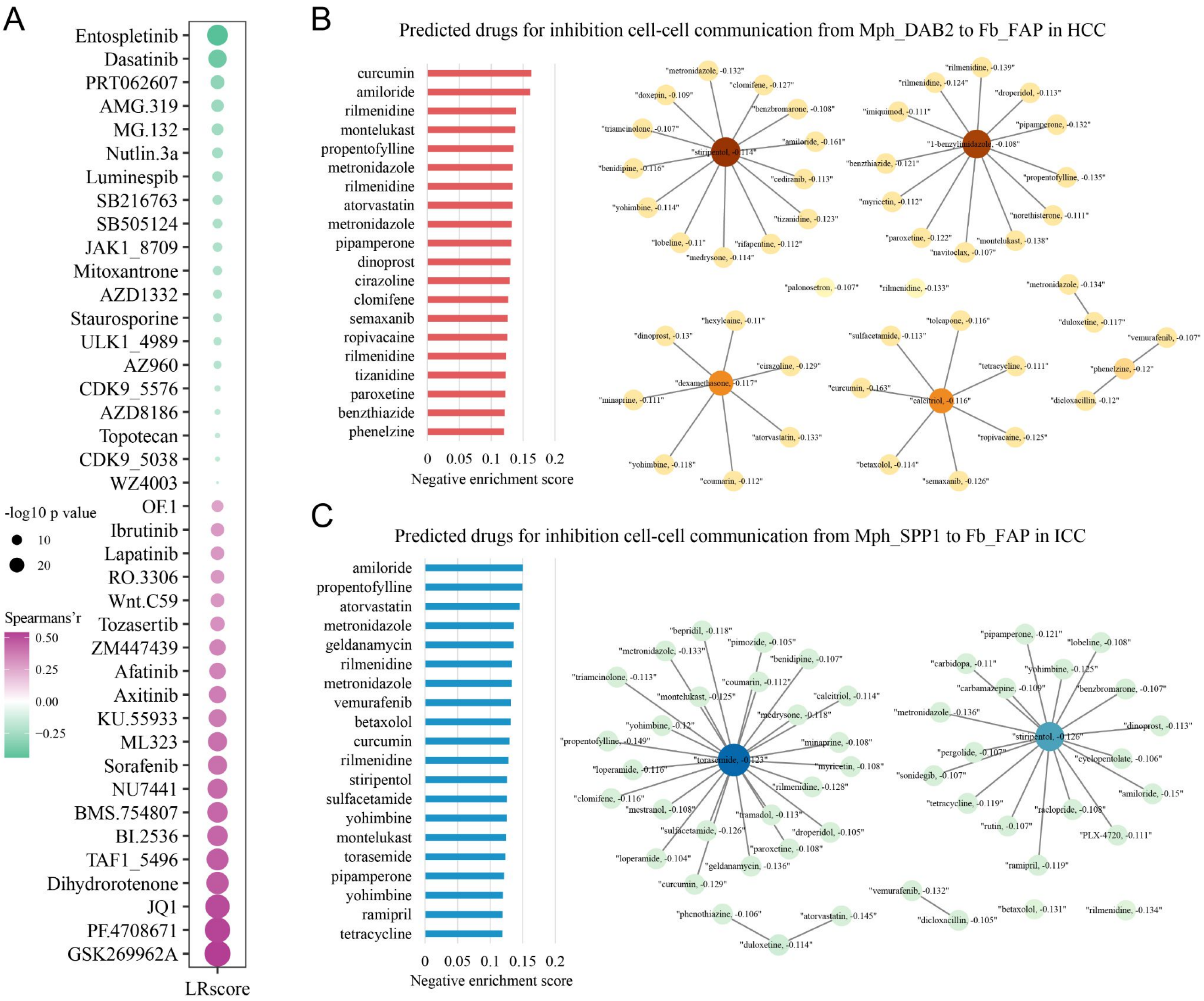

A
Entospletinib
Dasatinib
PRT062607
AMG.319
MG.132
Nutlin.3a
Luminespib
SB216763
SB505124
JAK1_8709
Mitoxantrone
AZD1332
Staurosporine
ULK1_4989
AZ960
CDK9_5576
AZD8186
Topotecan
CDK9_5038
WZ4003
OF.1
Ibrutinib
Lapatinib
RO.3306
Wnt.C59
Tozasertib
ZM447439
Afatinib
Axitinib
KU.55933
ML323
Sorafenib
NU7441
BMS.754807
BI.2536
TAF1_5496
Dihydrorotenone
JQ1
PF.4708671
GSK269962A
LRscore
-log10 p value
10
20
Spearmans'r
0.50
0.25
0.00
−0.25
B
Predicted drugs for inhibition cell-cell communication from Mph_DAB2 to Fb_FAP in HCC
curcumin
amiloride
rilmenidine
montelukast
propentofylline
metronidazole
rilmenidine
atorvastatin
metronidazole
pipamperone
dinoprost
cirazoline
clomifene
semaxanib
ropivacaine
rilmenidine
tizanidine
paroxetine
benzthiazide
phenelzine
0 0.05 0.1 0.15 0.2
Negative enrichment score
C
Predicted drugs for inhibition cell-cell communication from Mph_SPP1 to Fb_FAP in ICC
amiloride
propentofylline
atorvastatin
metronidazole
geldanamycin
rilmenidine
metronidazole
vemurafenib
betaxolol
curcumin
rilmenidine
stiripentol
sulfacetamide
yohimbine
montelukast
torasemide
pipamperone
yohimbine
ramipril
tetracycline
0 0.05 0.1 0.15 0.2
Negative enrichment score

Figure S12.

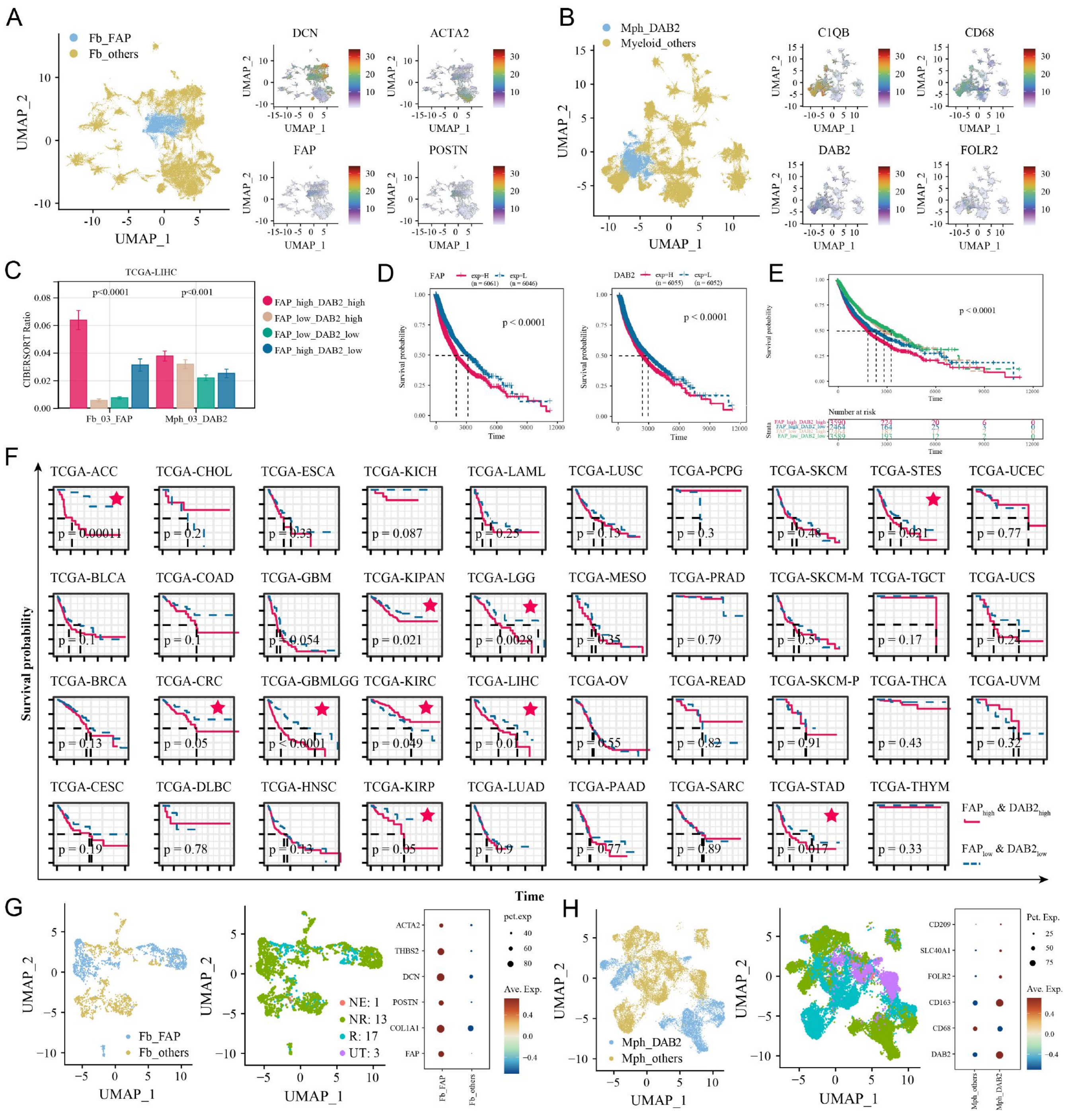
A
Fb_FAP
Fb_others
UMAP_1
UMAP_2
DCN
ACTA2
FAP
POSTN
B
Mph_DAB2
Myeloid_others
C1QB
CD68
DAB2
FOLR2
C
TCGA-LIHC
CIBERSORT Ratio
p<0.0001
p<0.001
FAP_high_DAB2_high
FAP_low_DAB2_high
FAP_low_DAB2_low
FAP_high_DAB2_low
Fb_03_FAP
Mph_03_DAB2
D
FAP
DAB2
Survival probability
Time
p < 0.0001
E
p < 0.0001
Number at risk
Strata
F
TCGA-ACC
TCGA-CHOL
TCGA-ESCA
TCGA-KICH
TCGA-LAML
TCGA-LUSC
TCGA-PCPG
TCGA-SKCM
TCGA-STES
TCGA-UCEC
TCGA-BLCA
TCGA-COAD
TCGA-GBM
TCGA-KIPAN
TCGA-LGG
TCGA-MESO
TCGA-PRAD
TCGA-SKCM-M
TCGA-TGCT
TCGA-UCS
TCGA-BRCA
TCGA-CRC
TCGA-GBMLGG
TCGA-KIRC
TCGA-LIHC
TCGA-OV
TCGA-READ
TCGA-SKCM-P
TCGA-THCA
TCGA-UVM
TCGA-CESC
TCGA-DLBC
TCGA-HNSC
TCGA-KIRP
TCGA-LUAD
TCGA-PAAD
TCGA-SARC
TCGA-STAD
TCGA-THYM
FAPhigh & DAB2high
FAPlow & DAB2low
Survival probability
Time
G
Fb_FAP
Fb_others
NE: 1
NR: 13
R: 17
UT: 3
ACTA2
THBS2
DCN
POSTN
COL1A1
FAP
pct.exp
Ave. Exp.
H
Mph_DAB2
Mph_others
CD209
SLC40A1
FOLR2
CD163
CD68
DAB2
Pct. Exp.
Ave. Exp.

Figure S13.

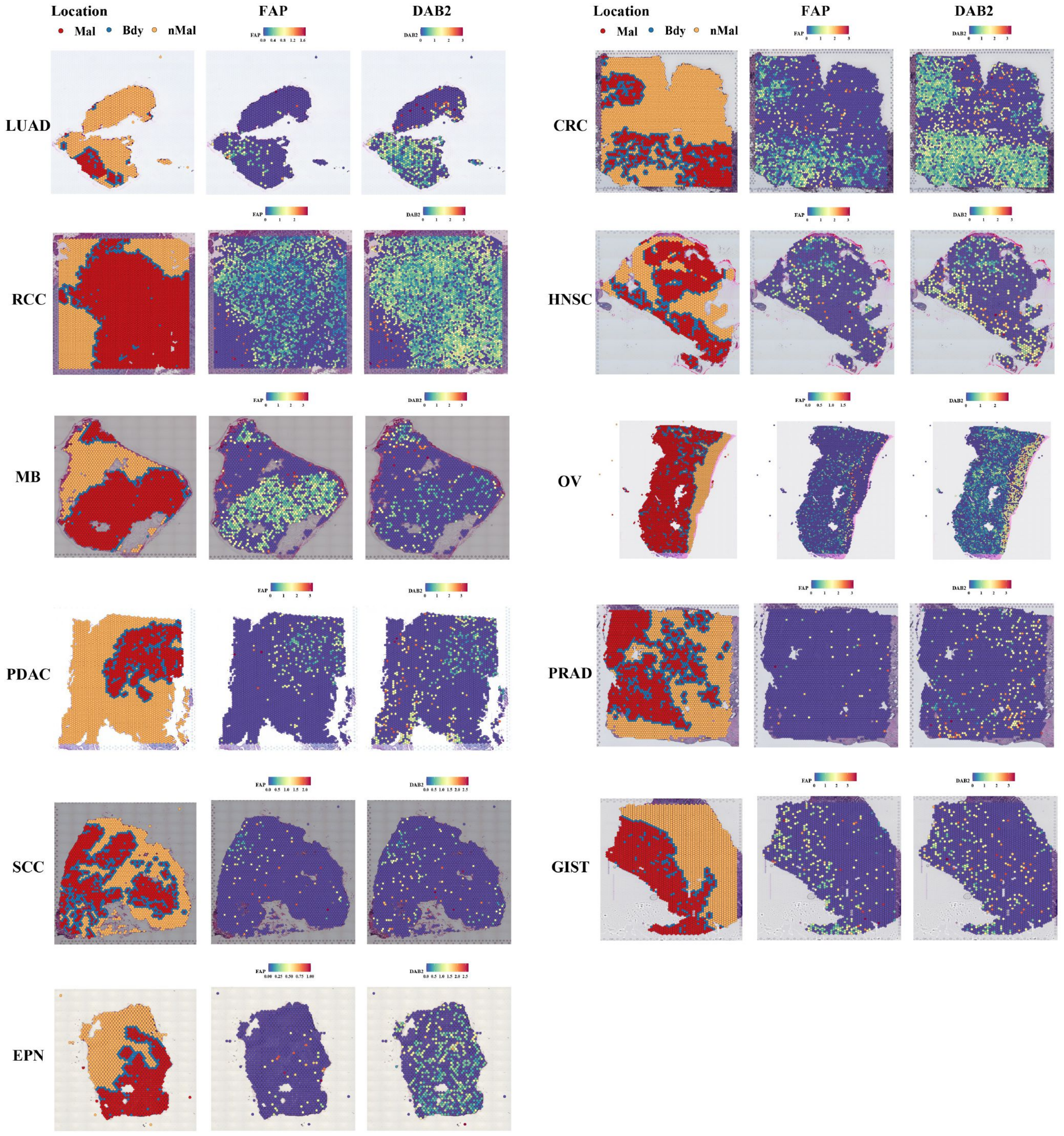
Location
Mal
Bdy
nMal
FAP
DAB2
LUAD
RCC
MB
PDAC
SCC
EPN
CRC
HNSC
OV
PRAD
GIST
